# Non-synonymous SNPs variants of PRKCG and its association with oncogenes predispose to hepatocellular carcinoma

**DOI:** 10.1186/s12935-023-02965-z

**Published:** 2023-06-21

**Authors:** Fizzah Abid, Khushbukhat Khan, Yasmin Badshah, Naeem Mahmood Ashraf, Maria Shabbir, Arslan Hamid, Tayyaba Afsar, Ali Almajwal, Suhail Razak

**Affiliations:** 1 Department of Healthcare Biotechnology, Atta-Ur-Rahman School of Applied Biosciences, National University of Sciences and Technology, Islamabad, 44010 Pakistan; 2 School of Biochemistry and Biotechnology, University of the Punjab, Lahore, 54590 Pakistan; 3 LIMES Institute (AG-Netea), University of Bonn, Carl-Troll-Str. 31, 53115 Bonn, Germany; 4 Department of Community Health Sciences, College of Applied Medical Sciences, King Saud University, Riyadh, Saudi Arabia

**Keywords:** Non-synonymous SNPs, PKC γ, *In-silico* tools, Therapeutics, Protein Kinase C

## Abstract

**Background:**

PRKCG encodes PKC γ, which is categorized under the classical protein kinase C family. No studies have specifically established the relationship between PRKCG nsSNPs with structural and functional variations in PKC γ in the context of hepatocellular carcinoma (HCC). The present study aims to uncover this link through in-silico and experimental studies.

**Methods:**

The 3D structure of PKC γ was predicted. Molecular Dynamic (MD) Simulations were run and estimates were made for interactions, stability, conservation and post-translational alterations between wild and mutant structures. The association of PRKCG levels with HCC survival rate was determined. Genotyping analyses were conducted to investigate the deleterious PRKCG nsSNP association with HCC. mRNA expression of PKC γ, HIF-1 alpha, AKT, SOCS3 and VEGF in the blood of controls and HCC patients was analyzed and a genetic cascade was constructed depicting these interactions.

**Results:**

The expression level of studied oncogenes was compared to tumour suppressor genes. Through Alphafold, the 3D structure of PKC γ was explored. Fifteen SNPs were narrowed down for in-silico analyses that were identified in exons 5, 10 and 18 and the regulatory and kinase domain of PKC γ. Root mean square deviation and fluctuation along with the radius of gyration unveiled potential changes between the wild and mutated variant structures. Mutant genotype AA (homozygous) corresponding to nsSNP, rs386134171 had more frequency in patients with OR (2.446), RR (1.564) and P-values (< 0.0029) that highlights its significant association with HCC compared to controls in which the wild genotype GG was found more prevalent.

**Conclusion:**

nsSNP rs386134171 can be a genetic marker for HCC diagnosis and therapeutic studies. This study has laid down a road map for future studies to be conducted on HCC.

**Supplementary Information:**

The online version contains supplementary material available at 10.1186/s12935-023-02965-z.

## Background

HCC originates from hepatocytes and accounts for 75 percent to 85 percent of primary liver cases [1, 2] [3] Its incidence is globally rising, primarily because of the growing prevalence of hepatitis B and C infections. Studies report that about 60%-70% of its cases are mainly linked with these two viruses [4]. Moreover, it is predicted that the incidence of HCC will further elevate after 2025, with cases increasing to 1 million per year [4].With regard to cancer related deaths, it is still a major contributor [5]. Also, it is not diagnosed early, which reduces its 2-year survival chances to less than 50%, while its 5-year survival rate is less than 10% in the US population [6]. Moreover, there is a need to explore the SNPs (Single Nucleotide Polymorphisms) that lead to its pathogenesis.

The most frequent mutations in humans are notified as SNPs and they contribute to only 0.1% of phenotypic differences when the genomes oftwo individuals arecompared within a population [7]. It is established that there are over a million SNPs in DNA coding regions and intronic or intergenic sequences that do not directly code for or translate into amino acids [8]. Moreover, SNPs can lead to different amino acids, which may affect the encoded protein function and the course of the disease [9]. The coding area is thought to include 50% SNPs, 25% of which are silent or synonymous SNPs, and 25% are missense SNPs [3]. The physiological or anatomical diversity of human proteins is driven by nsSNPs (non-synonymous SNPs), and multiple nsSNPs reconfigure the geneinteraction network through disease-associated proteins [5, 6]. Moreover, not all coding region SNPs are crucial in terms of functionality [10]. According to an estimate, about 20% of nsSNPs damage proteins [11]. It is inevitable to identify nsSNPs that participate in disease initiation by impacting proteins' conformation.

The current work is centered on nsSNP variants of the PRKCG gene, which is localized at chromosome 19q13.4.2 and encodes the classical PKC (Protein Kinase C) enzyme, i.e., PKCγ in humans [12]. According to the information gathered from ENSEMBL [13], the PRKCG gene is 3149 base pairs long,and PKCγ consists of 697 amino acids. Moreover, PKCγ comprises several varied regulatory domains in addition to a conserved kinase, catalytic domain that encloses the protein′s C-terminus. The C1A, C1B, and C2 make up PKCγ′s regulatory domain. Additionally, the regulatory domains of PKCγ include a related NH2 terminus and a pseudosubstrate (PS) motif. The C1 domain is split into the C1A and C1B domains, which are cysteine-rich domains, with the usual core structure including two histidines and six conserved cysteines, which work in coordination with two zinc ions [14]. There is mounting evidence that the PKC′s C1A and C1B domains play separate roles in the activation steps [15]. DAG (Diacylglycerol) andPMA (phorbol 12-myristate 13-acetate) bind to the C1 domain. The C2 domain depends on calcium to bind phospholipid molecules. The C3 and C4 domains perform the function of the kinase [16]. To fully activate the protein, three conserved sites on PKC are phosphorylated (P): T514 in the catalytic domain and T655 and T674 in the C-terminal tail [17]. The regulatory, kinase,and C-terminal tail work in conjunction to bring about stability in the PKCγ structure.

Different diseases have been confirmed with the PRKCG gene mutation [8, 11]. However, research on this gene, especially in the context of HCC, is limited, and no study has come to limelight that can associate nsSNPs of PRKCG with HCC pathogenesis. The current study delineated pathogenic nsSNPs in the PRKCG gene and investigated the effect of these nsSNPs on protein functional behavior in the context of HCC. We consulted *in-silico* tools at first, as they provide firm grounds based on which the results of wet-lab analysis could be predicted. Moreover, they are inevitable for initial analysis and constructing the roadmap for future therapeutic studies [18]. Through our wet-lab study, PRKCG expression along with the major genes involved in HCC, i.e. HIF1 *α,* AKT, SOCS3, and VEGF, was measured, and a pathway was constructed that interlinked these genes in different pathways. A genotyping analysis was also conducted, and the narrowed down non-synonymous SNP (rs386134171) association with HCC was determined. This is our second study in which we have identified a novel genetic marker of PRKCG, i.e., nsSNP rs386134171, through *in-silico* and experimental studies. Our first study [19] was also centered on HCC, where we reported a unique genetic marker. This study is different from our previous study, and in this study, we have included evolutionary conservation and Post Translational Modifications (PTMs) results, as well as survival and pathological stage wise analysis. To add to this, the PRKCG expression along with major genes involved in HCC i.e., HIF1 *α,* AKT, SOCS3 and VEGF was measured, and pathway was constructed interlinking these genes in different pathways.

## Methods

### In silico method

#### nsSNPs data collection

The PRKCG variant data (including rsIDs, chromosomal position, AA coordinates, and amino acid residue change) was fetched from DisGENET, ENSEMBL, COSMIC, ClinVar, and HGMD databases [20, 21]. Among all the SNPs present in PRKCG, the missense variants (under the coding category) with the transcript ID: ENST00000263431 were included, while the variants not provided with the rsIDs and those that were redundant were excluded in the final filtration step.

#### Pathogenicity Prediction of nsSNPs

The deleterious nsSNPs were distinguished from neutral (non-deleterious) ones by utilizing a bioinformatics tool scoring system [22]. For nsSNPs, SIFT, CADD, PolyPhen-2, REVEL, MetaLR, and Mutation Assessor were utilized [23]. nsSNP was considered pathogenic only if more than four tools predicted it to be deleterious through established criteria [24]. The selected nsSNPs were screened out and finalized for further analysis by defining a stringent criterion. Moreover, the selected nsSNPs were assessed through computational tools: FATHMM and PROVEAN [25], which employ specific algorithms to predict the effect of non-synonymous variants on the functional status of proteins. The scrutinized pathogenic nsSNPs were positioned on exons using genomic coordinates and exon number information gained from ENSEMBL.

#### PKC γ 3D structure determination

The 3D conformation aids in discerning a protein’s molecular function [26]. AlphaFold [27] was preferred to predict the PKC γ protein structure. AlphaFold uses an AI system to fold the input protein sequence into its corresponding structure. The 3D molecular viewer and AlphaFold work together to make it easier to recognize domains with corresponding locations and orientations The model confidence scores greater than 90, as predicted by AlphaFold, signify the higher accuracy of estimated residues position within the protein structure [28]. Additionally, InterProScan [29] was employed to predict the existence of significant functional sites and categorize protein sequences into domains. Entries from the family and domain that were organized into separate, non-overlapping hierarchies were examined as well.

#### Structural and functional alteration prediction of PKC γ nsSNP Variants

The structural variations of native and mutant proteins with changed ligand interactions were observed through PyMol software [30] through inserting desired single nucleotide mutations within the wild-typePKC γ structure. Because the protein structure of the variant rsIDs was not available, they were generated by utilizing the wild-type structure of PKC γ. The web application tool MutPred2 [31] predicted the molecular basis of disease and the processes underlying the disruption of protein conformation. For the automated analysis of mutants and analyses of point mutations on the structural conformation and function of proteins, Project HOPE software [32] was used.

#### nsSNPVariants Effect on

Protein Structure Stability: by either reducing or enhancing protein stability, SNPs frequently have an impact on protein stability [33]. The mutated protein structure’s stability status was checked, and comparison with the wild structure was made through a combination of analyses, including I-Mutant, MUpro, and DynaMut [34]. The stability differences were predicted through ΔΔG values.

#### PTMs identification of amino acid residues

PTM sites contain a variety of amino acid alterations that result in the production of a diverse range of proteins. These locations play a key role in cellular architecture and in processes including protein–protein interactions and disease-related signaling cascades [35]. Thus, predicting PTM information helps understand the effect of variations in terms of disease association or pathogenicity. PTM code2 (https://ptmcode.embl.de/) was visited for detecting the overall PTMs. PTM code2 predicts PTMs of input protein sequences and provides the result for 14 distinct PTMs.

#### Determination of evolutionary conservation of amino acid residues

Variants present in protein regions that are evolutionary conserved tend to disrupt the structure and function of a protein. The evolutionary conservation of amino acids was calculated using the ConSurf server [36]. The protein sequence in FASTA format was entered. The Homolog search algorithm selected was HMMER, the number of iterations was kept to 1, and the E-cut off value selected was 0.0001. The UNIREF-90 protein database was used for the homolog search. Next, the automatic operation was selected for ConSurf analysis. 150 sequences were selected for the analysis of the homolog search. The Maximal %ID between sequences was kept to a value of 95 whileminimal %ID for homologs was kept to a value of 35. For the alignment method, MAFFT-L-INS-i was selected,and the calculation method selected was Bayesian. The user-email ID was entered to retrieve the results through email.

#### Assessing structural variations with time through molecular dynamics simulations

For a clear depiction of structural variations with time, MD simulations of native and mutant structures (caused by nsSNPs) were run with the aid of GROMACS software [37]. Systems were solvated in a cube-shaped box with water molecules at a marginal radius of 1 nm, and electrical neutralization of the system was accomplished by introducing sodium (Na +) and chloride (Cl-) ions to the simulation box. For the energy minimization, the steepest descent minimization technique with an energy step size of 0.01 was used,and maximum numbers of iterations of 50000 steps were achieved. To provide a stable environment for the system, Berendsen temperature (tcouple) of 300 K and Parrinello-Rahman pressure (pcouple) of 1 bar were utilized. All electrostatic interactions were performed using the PME (Particle- Mesh Ewald) technique. Structures were equilibrated in NPT (pcoupl) and NVT (tcouple) for 100 ps. Finally, 20-ns MD simulation was run on both native and mutant structures. Once the required time of simulation was completed, the trajectory analysis command was entered, through which md_0_1_noPBC.xtc was generated. Next, the analyses were conducted utilizing this trajectory file. At first, anrmsd.xvg file was produced that identified the root mean square deviations, followed by rmsf.xvg that gave results for the root mean square fluctuations. Finally, gyrate.xvg was generated as the output file that identified the radius of gyration (Rg) of the input structures. The results obtained were plotted graphically.

#### Comprehending the clinical profile and survival analysis among HCC Patients

The gene expression differs when a comparison between normal and tumor samples is done [38]. Identifying the expression differences of genes at different stages can prove vital in patient diagnosis and prognosis [39]. PRKCG expression levels at different pathological stages and the survival analysis were done through OncoDB (http://oncodb.org.), which intakes clinical data from TCGA (The Cancer Genome Atlas) [40]. The cancer cases that are selected are categorized into high or low groups based on RNA expression levels, using a percentage cutoff. The survival plot generated provides the p-value and the hazard ratio from the log-rank test and Cox proportional regression analysis respectively [40].

## Experimental method

### Collection of blood samples

Samples of blood were obtained from controls with no history of disease and from patients confirmed with HCC diagnosis. Prior to collecting samples, patients and controls were informed ofthe purpose of the research and asked to sign a form of consent. About 100 controls and 100 patients volunteered to become part of the study. Both males and females were included in the study, with an age range of18-74. The blood was collected from the Combined Military Hospital (CMH), Rawalpindi. The research was initiated after ASAB, NUST Institute Review Board (IRB) approval and was done keeping in view the Declaration of Helinski principles [41]. From the collected blood, both RNA and DNA were isolated.

### RNA extraction, reverse transcription and amplification of transcriptome

The RNA extraction was done for expression analysis using the TRI reagent according to a defined protocol [42]. The RNA obtained was converted to cDNA (complementary DNA) through the FIREScript^®^ RT cDNA synthesis kit that was purchased fromSolis BioDyne. According to the defined concentrations in the manual, a master mix was added to the samples and kept at 65 degrees centigrade for 5 min, 42 degrees centigrade for 60 min,and 70 degrees centigrade for 10 min. The DNA presence was evaluated by the Nanodrop 2000 spectrophotometer (Thermo Scientific). For DNA amplification, the SYBER Green qRT-PCR (Real-Time Quantitative Reverse Transcriptase Polymerase Chain Reaction) Kit was used according to a defined protocol. For qRT-PCR, the primers for the genes (PRKCG, HIF1* α* (Hypoxia inducible factor alpha)*,* AKT (Ak strain transforming), SOCS3 (Suppressor of cytokine signaling 3), VEGF (vascular endothelial growth factor), and Beta(β) actin) amplified were retrieved through literature (Table 1). At the end, gel electrophoresis was done by preparing 1% Agarose gel for DNA band visualization. Bromophenol dye was used as the tracking dye, The PCR conditions included initial denaturation at 95 °C for 5 min, annealing at 65 °C for 60 min, and extension at 72 °C for 10 min. A plot was generated that provided the starting quantity of the template molecule on the x-axis against the CT (Cycle Threshold) on y-axis. Beta actin was used as an internal control and a reference gene for the calculation of fold change by the 2^−ΔΔCt^ method.

**Table 1:** RT-PCR primer sequences for PRKCG, Hif-1 alpha, VEGF, SOCS3, AKT and β actin

Genes	Primer sequences (5ʹ-3ʹ)	Tm (^o^C)	Ta (^o^C)	Product size	References
PRKCG	F: CCTTCTGCGACCACTGT R: GCTGCAGTTGTCAGCAT	54.8 53.4	58	557 bp	[38]
Hif 1 alpha	F: CATCAGCTATTTGCGTGTGAGGA R: AGCAATTCATCTGTGCTTTCATGTC	57.4 56.2	58	83 bp	[39]
VEGF	F:CCTGGTGGACATCTTCCAGGAGTAC R: GAAGCTCATCTCTCCTATGTGCTGG	62 60.8	61	196 bp	[39]
SOCS 3	F: ATTTCGCTTCGGGACTAGC R: AACTTGCTGTGGGTGACCAT	55 57.1	58	126 bp	[40]
AKT	F: TAGGCATCCCTTCCTTACAGC R: CACTGTCCCATCCGGCTTCA	56.4 59.7	55	114 bp	[41]
Beta actin	F:GGGGTGTTGAAGGTCTCAAA R:TGTCACCAACTGGGACGATA	55 55.7	57	165 bp	[42]

### Network construction

HIF-1 alpha [43], AKT [44] and VEGF [45] are included among the genes that are involved in cancer progression,while SOCS3 [46] acts oppositely to oncogenes as a tumor suppressor. Through utilizing qRT-PCR, the STRING database [47], and a thorough literature search, PRKCG was interconnected with the aforementioned genes in distinct pathways.

### DNA Extraction and mutation analysis

The DNA extraction was done according to the phenol/chloroform method [48]. To validate the presence of DNA within the sample, gel electrophoresis was carried out. For mutation or genotyping analysis at locus, rs386134171, ARMS-PCR (Amplification Refractory Mutation System-Polymerase Chain Reaction) was done. The PCR (Polymerase Chain Reaction) primers were designed computationally via Primer1 [49]. Two sets of primers, two outer and two inner primers were designed. The inner primers were SNP specific and used for detection. The product size of the two outer primers was 312;and the product size of the forward inner primer (G allele) was 175, while the product size of the reverse inner primer (A allele) was 193. Primer1 also gave respective annealing temperatures (Ta) of primers. The Tm (melting temperature) of primers was calculated using Oligo Analyzer 3.1 [50] (Table 2). 20 µl of reaction mixture was prepared in each PCR tube that contained 2 µl of DNA, 7 µl of Solis BioDyne Master Mix, 7 µl of nuclease free water,and 1 µl of each of the four primers. The conditions for ARMS-PCR were set as 95 °C for 5 min for initial denaturation, 35 cycles at 30 s for each temperature of 95 °C, 60 °C and72 °C and final extension for 7 min at 72 °C. Finally, the gel electrophoresis was performed with 2% agarose gel. Both the RT-PCR and ARMS-PCR end products were visualized under UV illuminator and digitally photographed by the GEL-DOC system.

**Table 2:** ARMS PCR primer sequences for PRKCG SNP (rs386134171, G/A)

SNP	Primer sequences (5ʹ-3ʹ)	Tm(^o^C)	Ta(^o^C)	Product size
rs386134171 (G/A)	Forward inner primer (G allele): 160 CTTCAGCTTCCTCATGGTTCTAGGAACAG 188	68 °C	60 °C	175 bp
	Reverse inner primer (A allele): 214 CAGGAATCCAACCTTCCCAAAACTTCT 188	68 °C		193 bp
	Forward outer primer (5ʹ—3ʹ): 22 TCTGGCTCTTTCTTTCTCCTTTCCACAG 49	68 °C		312 bp
	Reverse outer primer (5ʹ—3ʹ): 333 ACGGCCCAACCTAACCATATTTGAAGAT 306	68 °C		

### Statistical analysis

SPSS software [51] and Graph Pad Prism [52] were used in the end for statistical analysis. The significant level was determined through Probability (P) scores of < 0.05. The Fisher exact test and the Chi-square test were used for analyzing the results. Relative risk (RR) and Odds ratio (OR) were calculated along with the 95% confidence interval. OR or RR has values ranging from 0 to 1, with values greater than 1.0 indicating increased risk or association between the studied factor and the disease, while values less than 1.0 point towards decreased association. To add to this, a value equal to 1.0 shows no association [53].

## Results

### Identification of deleterious variants of PRKCG

**Fig. 1:**
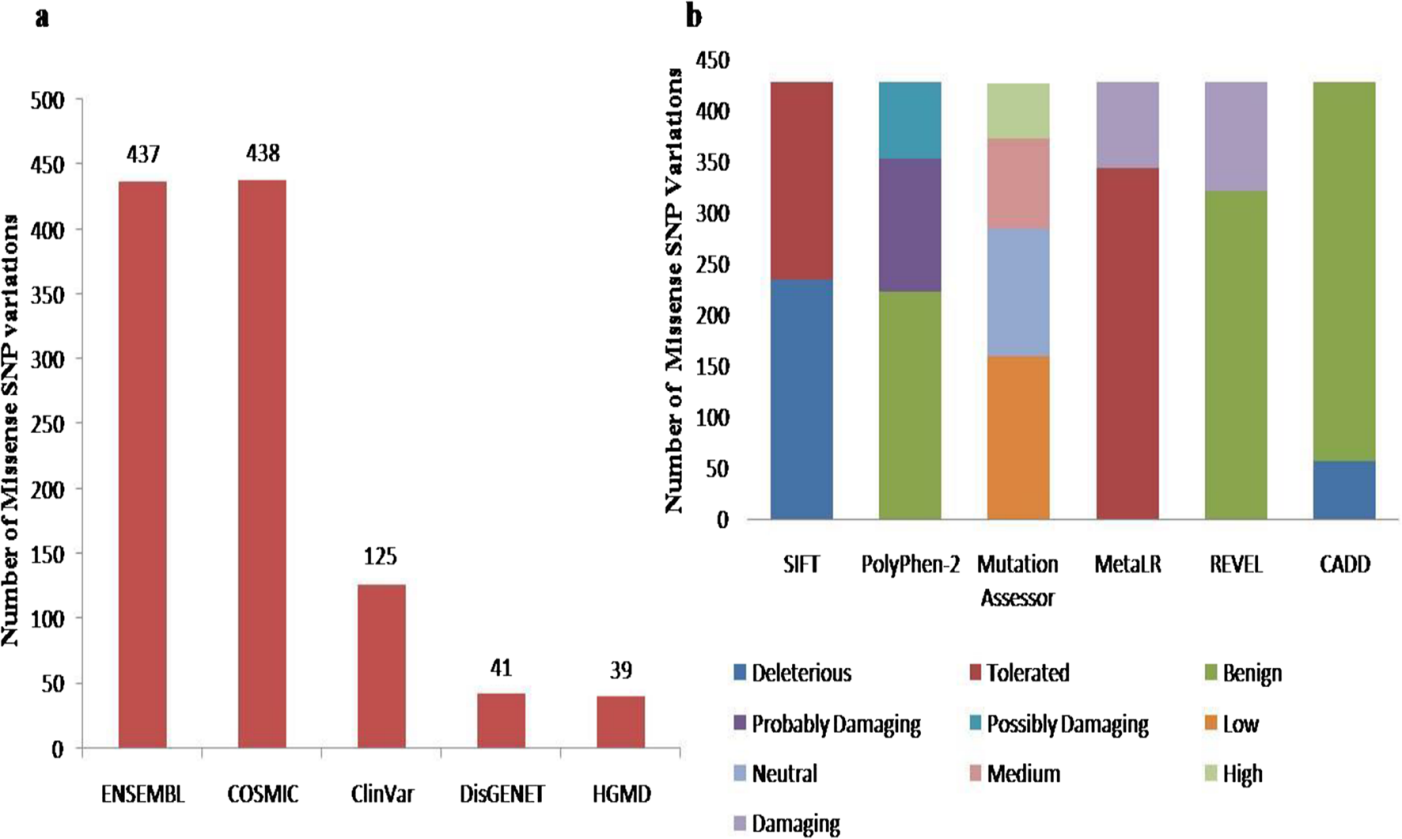
**a**. nsSNPs retrieved from six databases including ENSEMBL, COSMIC, ClinVar, DisGENET and HGMD. **b**. Filtration of nsSNPs as disease causing or non-deleterious through SIFT, PolyPhen-2, Mutation Assessor, CADD, REVEL and MetaLR

437 nsSNPs from ENSEMBL, 438 nsSNPs from COSMIC, 125 nsSNPs from ClinVar, 41 nsSNPs from DisGENET, and 39 nsSNPs from HGMD were obtained (Fig. 1a). So, the total nsSNPs gathered from different databases were 1080. After the filtration process, the final number of nsSNPs that remained was 427 (Additional file 1). The values obtained were validated from the dbNSFP database (http://sites.google.com/site/jpopgen/dbNSFP). The filtered rsIDs were sorted into deleterious or benign by utilizing bioinformatics SNP tools (Fig. 1b, Additional file 1).

Variants with SIFT scores that ranged from 0.00–0.05 were chosen as intolerant or deleterious, while those with scores greater than 0.05 were classified as tolerant or benign. Variants with PolyPhen-2 values of 0.25 were classified as benign; values between ‘ > 0.25 and  > 0.8’ were classified as ‘Possibly Damaging’, and values  > 0.8 were characterized as ‘Probably Damaging’. For mutation assessor scoring, variants with scores less than 0.8 were classified as neutral; scores > 0.8 but less than and equal to 1.9 were put in the low deleterious category; scores greater than 1.9 but equal to or less than 3.5 as medium deleterious; and scores greater than 3.5 as highly deleterious. The CADDs with scores equal to or greater than 30 were characterized as damaging. Moreover, the REVEL scores ranged from 0 to 1, with higher scores reflecting a greater likelihood that the variant was disease-causing. If the scores were less than or equal to 0.5, then the REVEL class was likely benign. Similarly, for MetaLR, scores less than or equal to 0.5 were taken as benign, while scores above 0.5 were considered damaging.

Moreover, the deleterious nsSNPs were narrowed down to 15 nsSNPs (Additional file 1). For that purpose, SIFT scores that were 0 or equal or less than 0.003 were selected; PolyPhen-2 scores above or equal to 0.975 were selected; CADD scores equal or above 30 were selected; and Mutation Assessor scores that were equal or above 0.862 were selected (Table 3). Variant IDs, chromosomal locations, substituted alleles, amino acids (AA), and AA coordinates of 15 nsSNPs were studied, and the data was collected (Table 4).

**Table 3:** The 15 filtered Variant IDs (nsSNPs) with bioinformatics SNP tools scores

Variant ID	Chr: bp	vf_allele	Alleles	AA	AA coord
rs797045900	19:53882648	A	T/A	C/S	52
rs386134162	19:53889693	A	G/A	C/Y	114
rs1064797249	19:53889695	T	G/T	D/Y	115
rs1568752939	19:53889734	C	G/C	G/R	128
rs386134167	19:53889744	A	G/A	C/Y	131
rs1192424800	19:53889900	A	G/A	V/M	138
rs1599943341	19:53889936	C	T/C	C/R	150
rs866406014	19:53889963	A	G/A	G/R	159
rs386134171	19:53898097	A	G/A	G/S	360
rs747522330	19:53898555	C	T/C	L/P	403
rs1398783758	19:53900448	C	T/C	L/P	468
rs387906679	19:53900612	T	G/T	D/Y	480
rs1202130595	19:53900736	G	A/G	Y/C	521
rs1568764306	19:53906342	A	G/A	R/H	597
rs121918516	19:53906728	C	T/C	F/L	643

**Table 4:** The 15 filtered nsSNPs with Variant IDs, Chromosomal Location, replaced alleles, amino acids (AA) and AA coordinates

Variant ID	SIFT	PolyPhen-2	CADD	REVEL	MetaLR	Mutation assessor
rs797045900	0	1	31	0.927	0.9962	4.825
rs386134162	0	0.988	31	0.967	0.997	4.7
rs1064797249	0	0.988	32	0.921	0.908	3.05
rs1568752939	0	0.993	32	0.936	0.947	4.65
rs386134167	0	0.988	32	0.937	0.997	4.715
rs1192424800	0	0.975	32	0.755	0.92	3.65
rs1599943341	0	0.999	32	0.907	0.944	4.735
rs866406014	0.003	1	32	0.662	0.74	3.97
rs386134171	0	0.993	31	0.978	0.961	3.73
rs747522330	0	1	32	0.789	0.276	3.02
rs1398783758	0	1	32	0.671	0.43	4.49
rs387906679	0	1	33	0.933	0.939	4.29
rs1202130595	0	1	32	0.687	0.405	3,62
rs1568764306	0	1	32	0.804	0.701	4.57
rs121918516	0	1	32	0.942	0.908	3.625

### Domains recognition

**Fig. 2:**
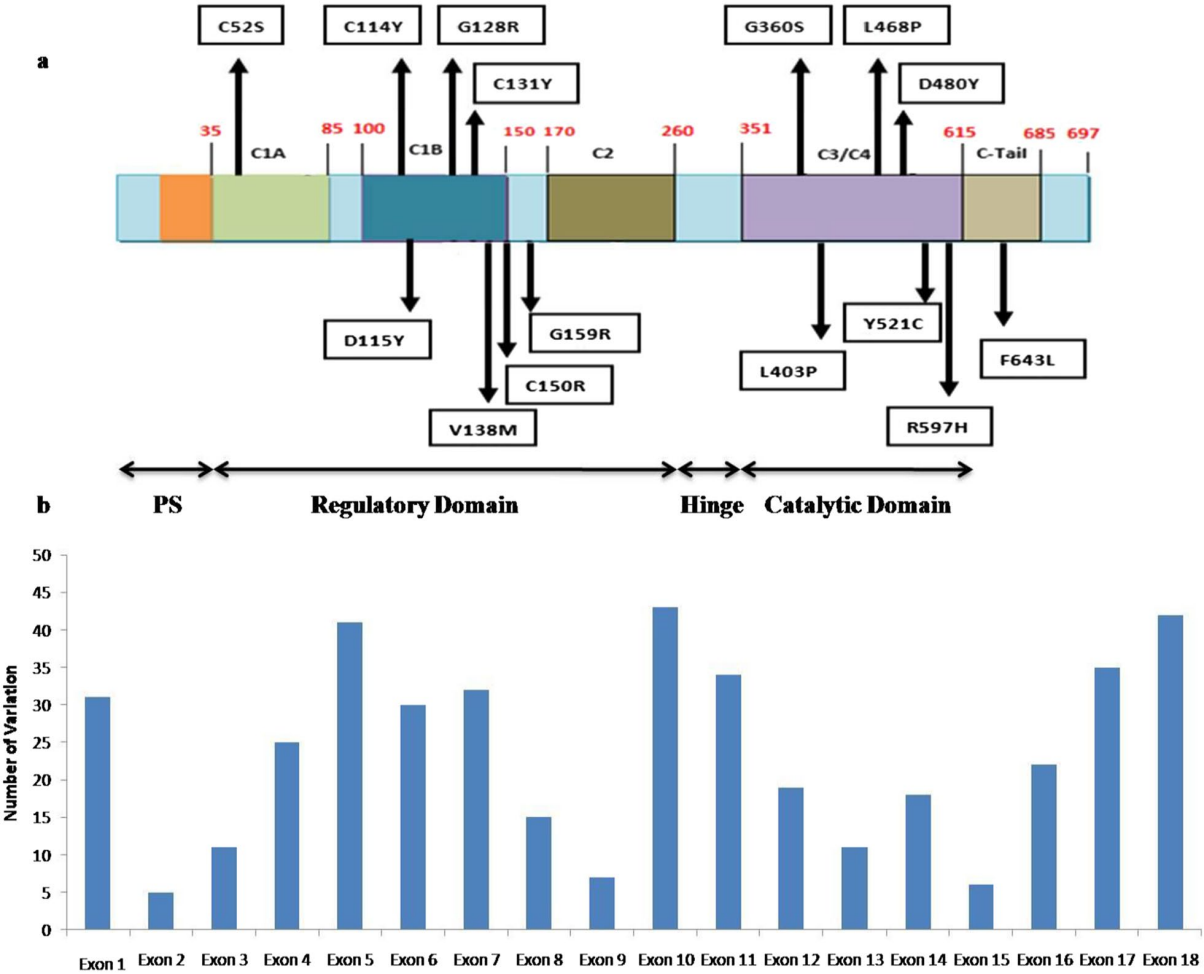
**a**. Domains and amino acid coordinates of PKC γ with 15 nsSNP variants. **b**. Distribution of 427 nsSNP variants that fall in different exon positions of PRKCG

Domains of PKC γ were predicted through InterProScan. The positions of corresponding amino acid residues for 15 nsSNPs were narrowed down (Fig. 2a). One residue was identified in the C1A region, six residues in the C1B region, one residue in the region between C1B and C2, six residues in the C3/C4 region, and one residue was found in the C-terminal tail. The pseudosubstrate domain was identified between 1 and 35 amino acid coordinates, the regulatory domain between 35 and 260 residues, the hinge region between 260 and 351 amino acids, the catalytic domain between 351 and 615 amino acids, and the C-terminal tail between 615–685 amino acids (Additional file 1). It can be seen that among 427 nsSNPs, the majority were identified in exons 5, 10, and 18 that gave rise to amino acid residue variations in different domains of PKCγ (Fig. 2b).

### PKCγ tertiary structure prediction

**Fig. 3:**
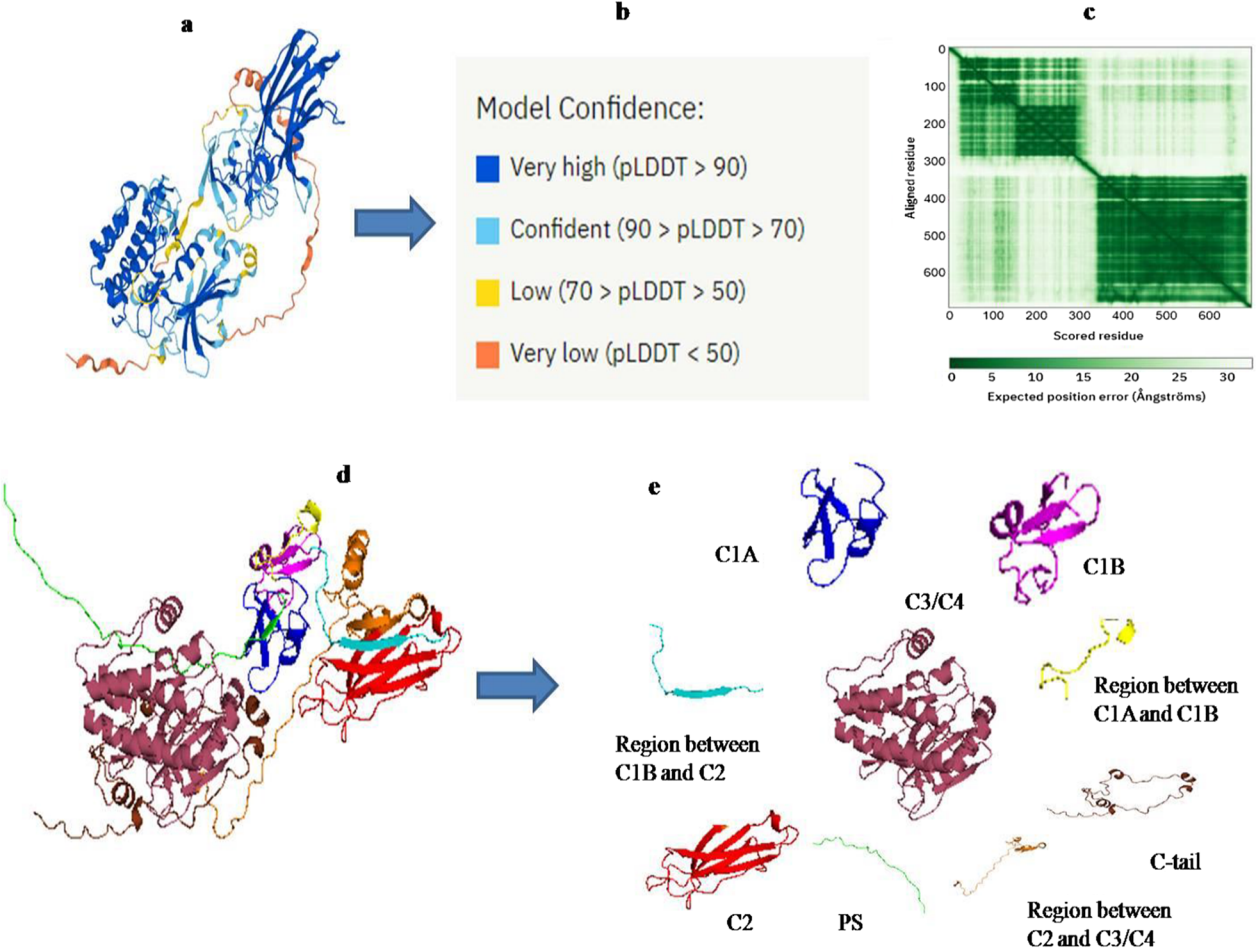
**a**. PKC γ 3D structure prediction by Alphafold. **b**. Confidence levels of the interactions found in the PKC γ structure. **c**. Expected position error of aligned residues of PKC γ. **d** and **e**. Elaborate structural view of domains of PKC γ that is drawn in conjunction with the information gained from Alphafold, InterProScan and PyMol. PKC gamma domains are represented in different colors, Pseudosubstrate (PS) region as green, C1A region as blue, region between C1A and C1B as yellow, C1B region as magneta, region between C1B and C2 as cyan), C2 (red), region b/w C2 and C3/C4 as orange), region C3/C4 as raspberry and C-tail as chocolate

PKC γ 3D structure was predicted through AlphaFold (Fig. 3a). The projected structure provided amino acid coordinates and confidence estimates for each residue on a scale from 0 to 100, with higher scores signifying greater confidence. The very high model confidence residues have pLDDT values ≥ 90. The residues were marked as confident with 90 > pLDDT ≥ 70 and residues with 70 > pLDDT ≥ 50 were marked as low confidence residues, while very low confidence residues had pLDDT < 50 (Fig. 3b). The model confidence levels at different amino acid positions of PKC γ were also observed (Additional file 1). Positional errors of aligned residues were analyzed as well (Fig. 3c). The expected position error is minimal in residues highlighted in dark green, while as the color turns towards light green, the expected position error increases, which highlights the unreliability of the relative orientation and position of residues within the protein structure. The PDB structure of PKC γ was retrieved from Alphafold and analyzed in PyMol. The domain information was obtained from InterProScan. Different domains of PKC γ are highlighted separately in different colors (Fig. 3d, e).

### Evolutionary analysis prediction

**Fig. 4:**
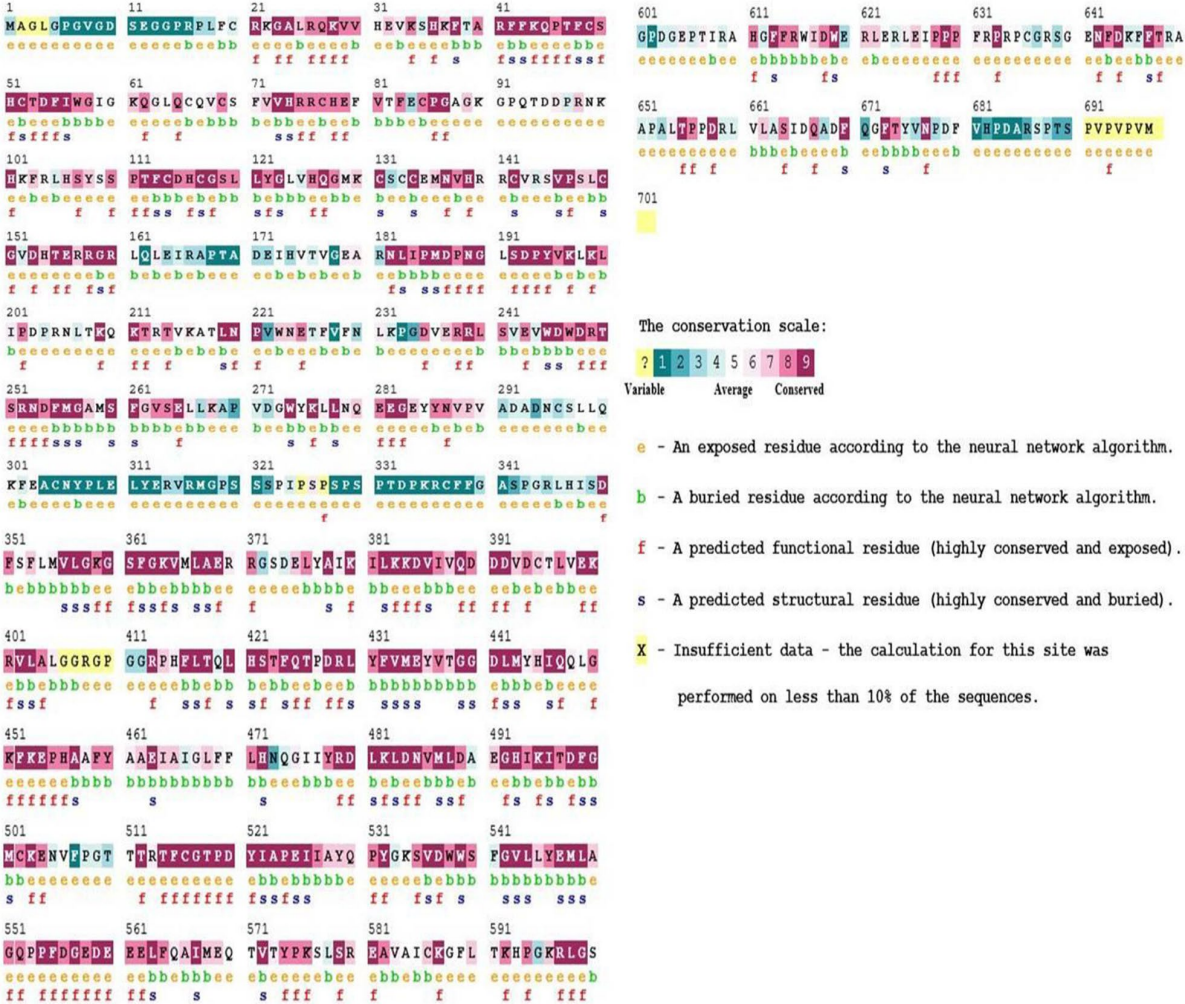
ConSurf analysis of PKC γ. Illustration of the evolutionary conservation scale and representation of the PKC γ residues as buried (b), exposed (e), functional (f), and structural (s)

ConSurf estimated the evolutionary conservation of amino acid positions in a protein molecule based on the phylogenetic relations between homologous sequences. Only the FASTA sequence of the protein was input at the beginning to retrieve the expected results. ConSurf gave an accurate evolutionary rate by using either an empirical Bayesian method or a maximum likelihood (ML) method. The ConSurf results highlighted the preservation sequence homology and conservation scores from 1 to 9 (Fig. 4), where residues with the maximum score, i.e., 9, were highly conserved, while residues with scores less than 5 were variable and less conserved. Moreover, the most variable positions (grades 1–4) were colored turquoise, intermediate or average conserved positions (grade 5) were colored white, and the most conserved positions (grades 6–9) were colored maroon (Fig. 4). Conservation scores obtained for PKC γ residues indicated that all 15 residues lay in positions that were highly conserved, so the mutations would expectably produce a damaging effect on the functionality and structural stability of the protein. According to ConSurf, residues G360, D480, Y521, and R597 were found exposed, while the rest of the residues were found buried within the protein structure (Fig. 4 and Table 5).

**Table 5:** The 15 filtered residue positions with their corresponding ConSurf values

Position	Residues	Scores of conservation	Color	Conservation levels	Buried/exposed (B/E)	Functional/structural role
52	C	− 0.818	9	Highly conserved	Buried	Structural
114	C	− 0.822	9	Highly conserved	Buried	Structural
115	D	− 0.665	9	Highly conserved	Buried	Structural
128	G	− 0.741	9	Highly conserved	Buried	Structural
131	C	− 0.822	9	Highly conserved	Buried	Structural
138	V	− 0.725	9	Highly conserved	Buried	Structural
150	C	− 0.823	9	Highly conserved	Buried	Structural
159	G	− 0.823	9	Highly conserved	Buried	Structural
360	G	− 0.656	8	Highly conserved	Exposed	Functional
403	L	− 0.719	9	Highly conserved	Buried	Structural
468	L	− 0.484	8	Highly conserved	Buried	–
480	D	− 0.842	9	Highly conserved	Exposed	Functional
521	Y	− 0.777	9	Highly conserved	Exposed	Functional
597	R	− 0.797	9	Highly conserved	Exposed	Functional
643	F	− 0.714	9	Highly conserved	Buried	Structural

### Sorting and filtration process through involving different bioinformatics tools

For initial sorting, SIFT, PolyPhen-2, REVEL, MetaLR, CADD, and Mutation Assessor were used. Then further bioinformatics tools were applied to the 15 filtered nsSNPs, which included FATHMM and PROVEAN. These tools were employed to further validate the filtered nsSNPs to determine whether they were deleterious or non-deleterious and had an effect on the biological activity or functional status of proteins. The associated nsSNV was projected as ′Damaging (D) if the FATHMM score was = − 1.5 (or rankscore >  = 0.81332) and the PROVEAN score was <  = − 2.5 (rankscore >  = 0.54382); otherwise, it was predicted as ′Tolerated (T) or ′Neutral (N) (Table 6). The molecular mechanisms of disruption brought about by structural or functional changes because of mutations were investigated through MutPred (Table 7).

**Table 6:** Assessing the pathogenicity of filtered nsSNPs through different softwares

Residues	FATHMM_score	FATHMM_converted_rankscore	FATHMM_pred	PROVEAN_score	PROVEAN_converted_rankscore	PROVEAN_pred
C52S	− 7.56	0.9989	D	− 7.62	0.95394	D
C114Y	− 7.56	0.9989	D	− 10.17	0.98905	D
D115Y	− 3.21	0.93291	D	− 8.14	0.96736	D
G128R	− 3.46	0.94508	D	− 7.13	0.94048	D
C131Y	− 7.56	0.9989	D	− 9.82	0.98684	D
V138M	− 3.54	0.94799	D	− 2.38	0.52451	N
C150R	− 3.27	0.98487	D	− 10.9	0.99248	D
G159R	− 0.8	0.91589	T	− 7.44	0.94872	D
G360S	− 3.98	0.96277	D	− 5.38	0.84954	D
L403P	1.3	0.3559	T	− 6.75	0.92692	D
L468P	0.73	0.50721	T	− 6.38	0.911	D
D480Y	− 3.16	0.92996	D	− 8.58	0.9743	D
Y521C	0.37	0.57729	T	− 8.59	0.97447	D
R597H	− 0.57	0.71307	T	− 4.22	0.75854	D
F643L	− 3.05	0.92407	D	− 5.54	0.86149	D

**Table 7:** MutPred2 conservation scores and the underlying molecular mechanisms disrupted because of alteration in amino acid residues

Residues	Conservation scores (g-value)	Molecular mechanisms disruption					
		Functional impact	Pr-value	P-value	Structural impact	Pr-value	P-value
		(Probability)			(Probability)		
C52S	0.960	Altered Transmembrane protein; Altered Metal binding; Gain of Disulfide linkage at C49	0.29 0.28 0.12	3.7e-04 0.02 0.04			
C114Y	0.955	Altered Metal binding; Altered Transmembrane protein; Gain of Sulfation at C114	0.56 0.12 0.02	2.9e-03 0.03 0.04			
D115Y	0.932	Altered Metal binding; Altered Transmembrane protein; Gain of Sulfation at D115	0.56 0.09 0.01	2.9e-03 0.05 0.04			
G128R	0.869	Altered Metal binding	0.29	0.02			
C131Y	0.901	Altered Metal binding; Gain of Pyrrolidone carboxylic acid at Q127	0.41 0.04	6.1e-03 0.05	Gain of Strand	0.26	0.04
V138M	0.431	-	-	-			
C150R	0.970	Altered Metal binding; Altered Transmembrane protein; Loss of Disulfide linkage at C150	0.55 0.11 0.22	3.0e-03 0.04 0.01	Gain of Intrinsic disorder; Altered Disordered interface	0.35 0.22 0.15	0.02 0.01 0.05
G159R	0.741	Altered Transmembrane Protein	0.13	0.02	Gain of Helix	0.32	2.2e-0.3
G360S	0.908	Loss of Acetylation at K364; Altered DNA binding; Loss of Methylation at K364; Loss of Catalytic site at K359	0.24 0.20 0.15 0.14	0.02 0.02 0.02 0.03			
L403P	0.971	Gain of Allosteric site at K400	0.23	0.02	Gain of Intrinsic disorder	0.35	0.02
L468P	0.932	Altered Metal binding; Gain of Allosteric site at H472; Altered Transmembrane protein; Altered DNA binding	0.31 0.26 0.14 0.14	2.2e-03 9.1e-03 0.02 0.05	Altered Ordered interface;	0.28	0.04
D480Y	0.946	Altered Metal binding; Loss of Allosteric site at R479; Altered DNA binding; Altered Transmembrane protein; Loss of Catalytic site at D484; Gain of Sulfation at D480	0.77 0.31 0.23 0.15 0.14 0.01	5.4e-05 3.5e-03 0.01 0.01 0.03 0.04	Altered Ordered interface; Altered Disordered interface;	0.32 0.28	1.8e-03 0.04
Y521C	0.945	Altered Metal binding; Gain of Catalytic site at C516; Loss of Allosteric site at Y521; Altered Transmembrane protein; Gain of Disulfide linkage at C516; Loss of Sulfation at Y521;	0.81 0.37 0.33 0.30 0.16 0.04	5.7e-04 2.8e-04 1.9e-03 1.3e-04 0.03 0.01	Altered Ordered interface; Altered Disordered interface; Gain of Relative solvent accessibility	0.44 0.29 0.26	5.7e-04 0.02 0.03
R597H	0.771	Altered DNA binding; Loss of Allosteric site at R597; Loss of Acetylation at K592; Loss of Amidation at P594	0.37 0.37 0.25 0.01	6.8e-04 9.2e-04 0.01 1.2e-03			
F643L	0.865	Loss of Acetylation at K645	0.40	1.3e-03	Altered Ordered interface; Gain of Helix	0.29 0.27	0.03 0.04

### Project hope analysis of protein structure and mutant analysis

With the help of Project Hope, the changes in the 3D tertiary structure of the protein and the associated functional or phenotypic changes were studied. It can be seen that those mutant amino acid residues that are bigger than the wild-type residues have become the primary cause of repulsions and bumps in the protein structure that have led to perturbed folding and the loss of essential hydrogen and hydrophobic interactions (Table 8).

**Table 8:** Project Hope Prediction Analysis

Residues	Hydrophobicity	Domain	Size	Charge	Conservation site	Amino acid Properties
C52S	Wild > Mutant	C1A	-	-	Highly Conserved	Perturbed folding; Loss of Hydrophobic interactions
C114Y	Wild > Mutant	C1B	Wild < Mutant	-	Highly Conserved	Bumps/repulsions of ligands; Loss of Hydrophobic interactions; Disrupted folding
D115Y	Wild < Mutant	C1B	Wild < Mutant	Wild-Negative, Mutant-Neutral	Highly Conserved	Loss of hydrogen bonds; Disrupted correct folding; Bumps/repulsions of ligands
G128R	Wild > Mutant	C1B	Wild < Mutant	Wild- Neutral, Mutant-Positive	Highly Conserved	Bumps/repulsions of ligands; Disrupted folding interactions
C131Y	Wild > Mutant	C1B	Wild < Mutant	-	Highly Conserved	Bumps/repulsions of ligands; Disrupted folding interactions; Loss of Hydrophobic interactions
V138M	-	C1B	Wild < Mutant	-	Highly Conserved	Bumps/repulsions of ligands; Disrupted folding interactions
C150R	Wild > Mutant	C1B	Wild < Mutant	Wild-Neutral,Mutant-Positive	Highly Conserved	Bumps/repulsions of ligands; Disrupted folding interactions; Loss of Hydrophobic interactions
G159R	Wild > Mutant	Between C1B and C2	Wild < Mutant	Wild-Neutral Mutant-Positive	Highly Conserved	Bumps/repulsions of ligands; Disrupted folding interactions
G360S	-	C3/C4	Wild < Mutant	-	Highly Conserved	Bumps/repulsions of ligands; Disrupted folding interactions
L403P	-	C3/C4	Wild > Mutant	-	Highly Conserved	Bumps/repulsions of ligands; Disrupted folding interactions
L468P	-	C3/C4	Wild > Mutant	-	Highly Conserved	Bumps/repulsions of ligands; Disrupted folding interactions
D480Y	Wild < Mutant	C3/C4	Wild < Mutant	Wild-Negative Mutant- Neutral	Highly Conserved	Loss of hydrogen bonds; Perturbed folding
Y521C	Wild < Mutant	C3/C4	Wild > Mutant	-	Highly Conserved	Bumps/repulsions of ligands; Disrupted folding interactions; Loss of hydrogen bonds
R597H	-	C3/C4	Wild > Mutant	Wild- Positive Mutant-Neutral	Highly Conserved	Loss of ionic interactions; Disrupted folding
F643L	-	C-Tail	Wild > Mutant	-	Highly Conserved	Loss of hydrophobic interactions; Disrupted folding

### Protein stability analysis

**Fig. 5:**
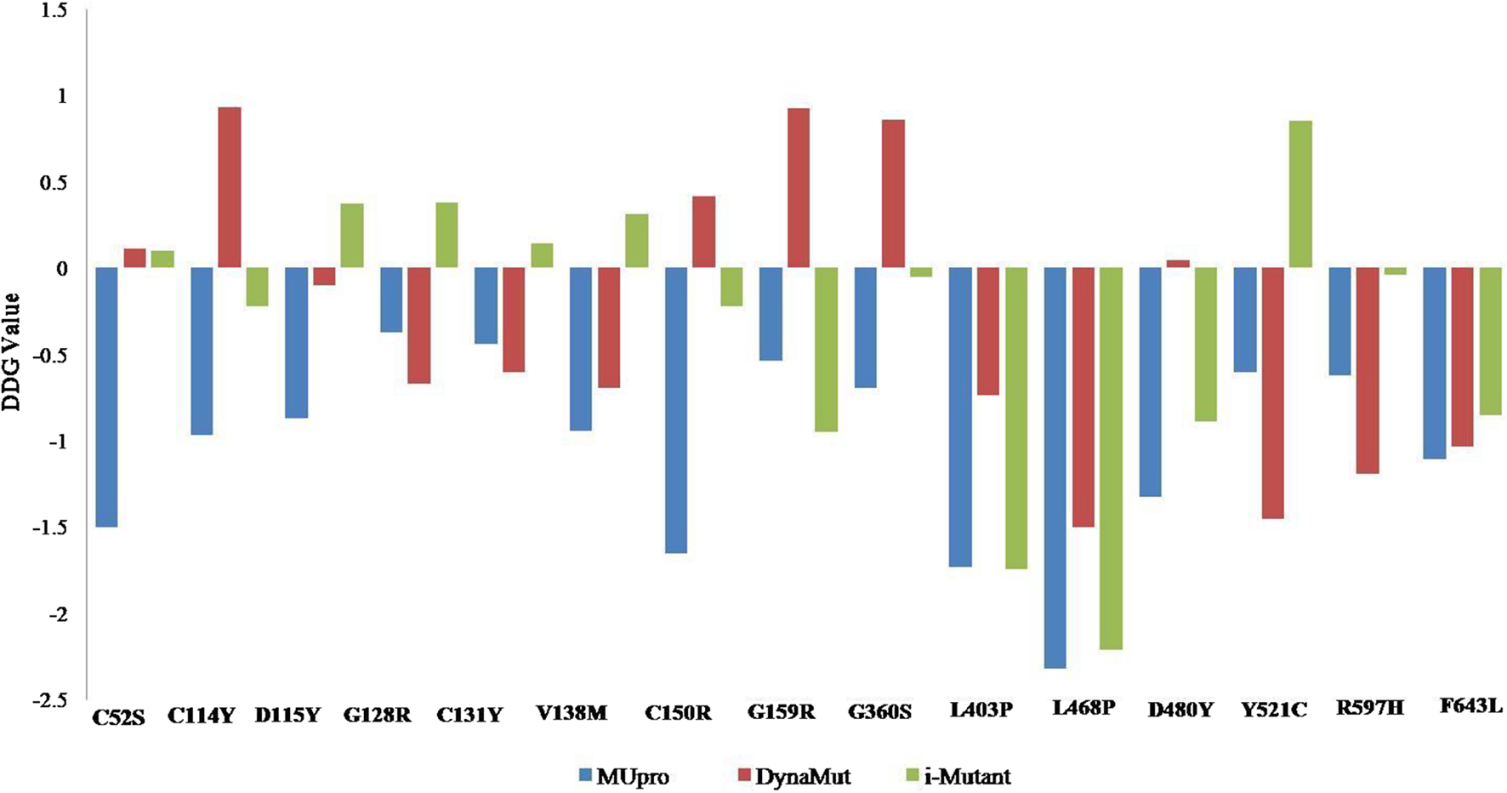
nsSNP stability analysis of PKC γ variants through MUpro, I-Mutant v2.0 and DynaMut

For protein stability analysis, the I-Mutant 3.0 gave the output as a Delta Delta G (DDG) value that gave three predictions: largely unstable (DDG <  − 0.5 kcal/mol), largely stable (DDG > 0.5 kcal/mol), or neutral (− 0.5 ≤ DDG ≤ 0.5 kcal/mol). The values that were less than 0.5 were assigned a negative value, while the values that were above 0.5 were assigned positive values. For MUpro, the same criteria were used to predict protein stability, a score less than 0 means the mutation decreases protein stability. Conversely, a score higher than 0 means the mutation increases the protein's stability. The higher the score, the more confident the prediction is. Moreover, in DynaMut, *DDG* was used as a metric for predicting how a single point mutation would affect protein stability. The more negative a value, the less stable the protein is, and the more positive the value, the more stable the protein is (Fig. 5, Additional file 1).

### PTM code2 for PTM prediction

The PTMs for the wild type PKC γ amino acid residue positions were identified (Additional file 1). The conservation scores of PKC γ residue positions highlighted the possibility that alterations brought about by the mutations could have a negative impact on the functional status of proteins due to altered modifications at the post translational level.

### Flexibility and molecular dynamics simulation analysis of mutants in regulatory domain of PKCγ

**Fig. 6:**
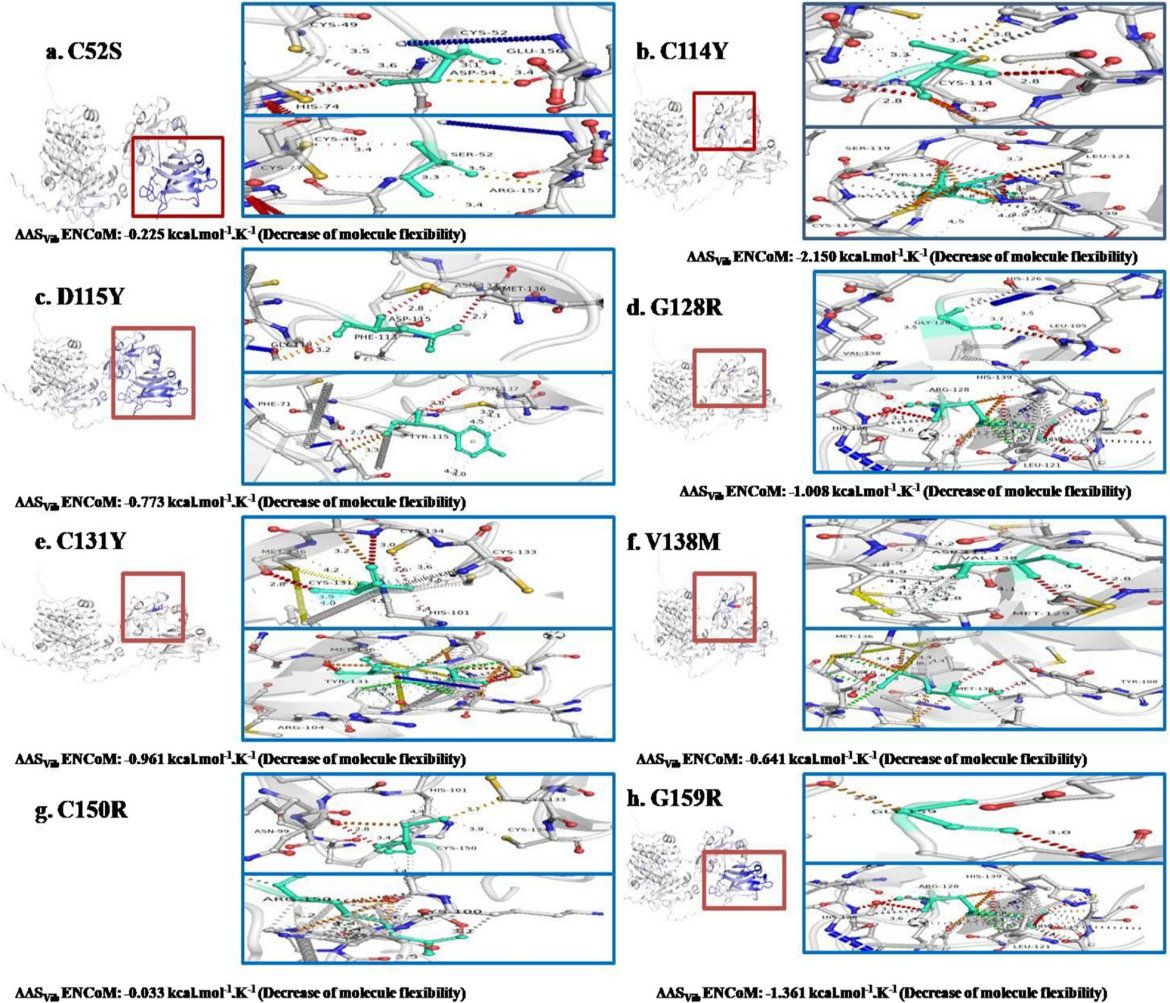
DynaMut results depiction of molecular flexibility analysis and interatomic interactions of regulatory domain of PKC γ. **a**. Variant C52S is shown. Hydrogen bonds are present at 3.1A-3.6A. **b**. Variant C114Y is shown. Hydrogen bonds are present between 2.8A and 3.5A (Angstrom) while hydrophobic interactions are displayed between 2.9A and 4.5A. The aromatic interactions are observed between 2.7A and 3.9A. **c**. Variant D115Y is represented. Hydrogen bonds are present between 2.7A to 3.2A. Hydrophobic interactions are observed at 3.9A and 4.5A. **d**. Variant G128R is shown. Hydrogen bonds are present between 2.4A and 3.7A. Hydrophobic interactions are displayed between 3.9A and 4.5A. **e**. Variant C131Y is shown. Hydrogen bonds are present between 2.8A and 3.7A. Hydrophobic interactions and aromatic interactions are observed at 3.0A-4.5A and 3.4A-3.9A respectively. **f**. Variant V138M is shown. Hydrogen bonds are present between 2.8A and 2.9A distance. Hydrophobic interactions are observed between 3.5A and 4.5A. **g**. Variant C150R is shown. Hydrogen bonds are present between 2.8A and 3.9A. Hydrophobic interactions are observed at 3.4A, 4.3A and 4.4A. **h**. Variant G159R is shown. Hydrogen bonds are present at 2.5A–3.0A. Hydrophobic interactions are observed between 3.8A and 4.5A. The region where mutation is present is highlighted inside the red box that depicts the rigidification of the structure and decrease of molecular flexibility in the represented structures

**Fig. 7:**
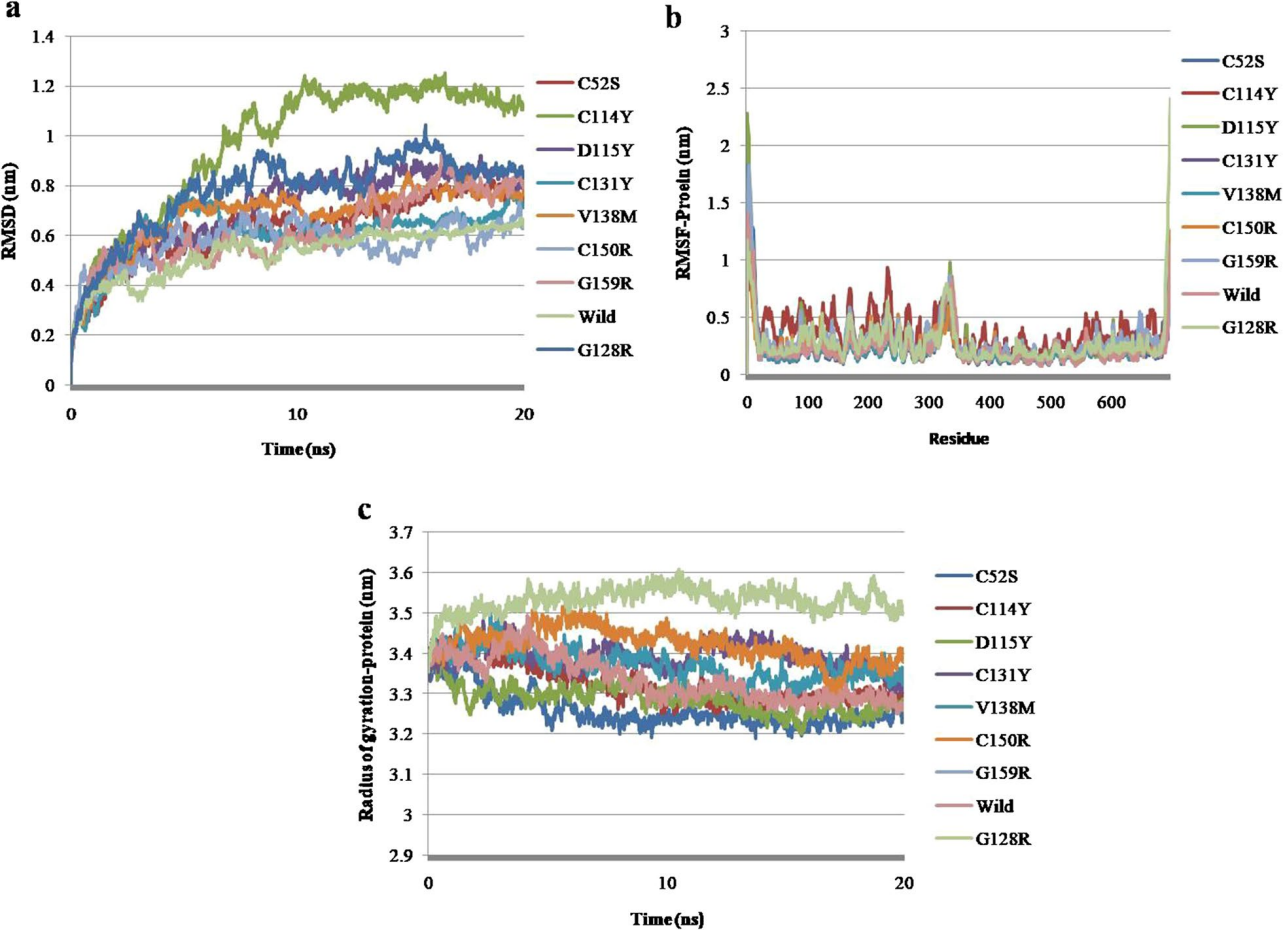
Molecular dynamics simulations representation of PRKCG nsSNPs residing in regulatory domain. **a**. Root mean square deviation (RMSD). **b**. Root mean square fluctuation (RMSF)—protein. **c**. Radius of gyration—backbone

C1A, C1B, and C2 constitute the PKC γ regulatory domain. Of the fifteen nsSNPs, the C1 domain harbored seven mutations (C52S, C114Y, D115Y, G128R, C131Y, V138M, and C150R). Moreover, all seven mutations were located within the C1B region, which caused the flexibility of the PKC **γ** to decrease in the region where the mutations were identified. The changes in inter-residue hydrogen bond interactions have been observed in C52S (Fig. 6a). In mutant residues, D115Y, G128R, V138M, and C150R, the changed hydrogen bond and hydrophobic interactions were identified (Fig. 6c, d, f, and g). In the case of C114Y, C131Y, along with changed hydrogen and hydrophobic interactions, there has been a gain in aromatic interactions (Fig. 6b and e). Between the regions C1 and C2, a single mutation, G159R, was identified that resulted in changed hydrogen bonds and hydrophobic interactions (Fig. 6h). A molecular dynamics simulation investigation showed that the mutants, C52S, C114Y, D115Y, G128R, C131Y, V138M, and G159R had increased RMSD values when compared with the wild type. The C150R mutant had RMSD values close to the wild type but showed fluctuation throughout 20 ns, which represents the instability of the C150R mutant structure (Fig. 7a). The fluctuations in RMSF values of mutants in the regulatory domain also indicate destabilization in the regions where the mutations were identified (Fig. 7b). Fluctuations in the radius of gyration were seen for the mutants C52S, C114Y, D115Y, G128R, C131Y, V138M, C150R, and G159R over the span of 20 ns that indicate the structural instability and loss of compactness of the PKC γ regulatory region (Fig. 7c).

### Flexibility and molecular dynamics simulation analysis of mutants in kinase domain of PKC γ

**Fig. 8:**
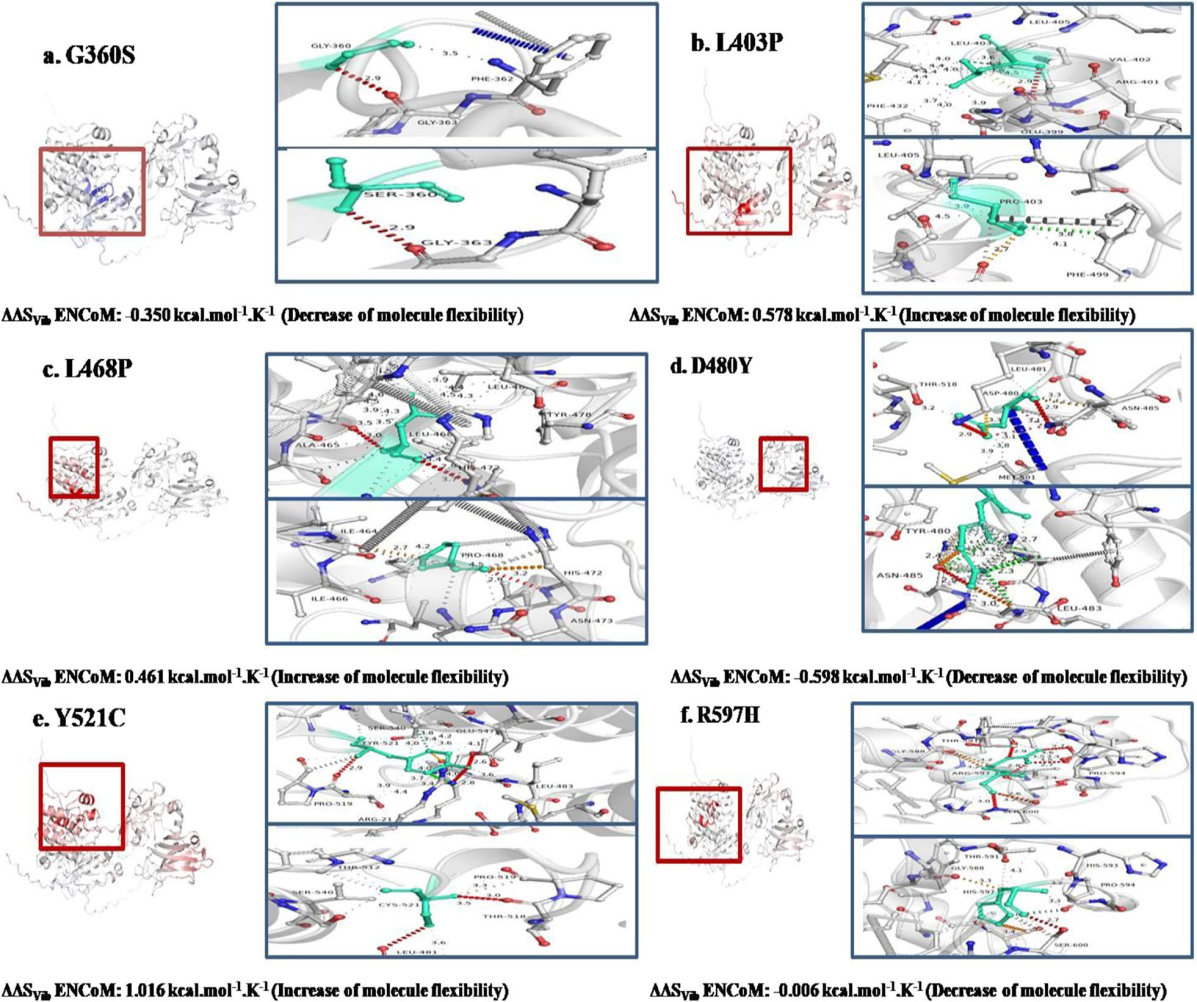
DynaMut tool depiction of molecular flexibility analysis and interatomic interactions of kinase domain of PKC γ. **a** Variant G360S is shown. Hydrogen bonds are present at 2.9A and 3.5 A distance. The region where mutation is present is highlighted with blue region inside the red highlighted box that depicts the rigidification of the structure. **b** Variant L403P is shown. Hydrogen bonds are present at 2.7A and 2.9A. The hydrophobic interactions are observed at 3.6A-4.5A. **c** Variant L468P is shown. Hydrogen bonds are present between 2.9A and 3.7A while hydrophobic interactions are observed at 3.9A-4.5A. **d**. Variant D480Y is shown. Hydrogen bonds are present between 2.9A and 3.3 and hydrophobic interactions are represented at 2.3A-3.9A. **e**. Variant Y521C is shown. Hydrogen bonds are present between 2.6A and 3.6A while the hydrophobic interactions are observed at 3.4–4.4A. **f**. Variant R597H is shown. Hydrogen bonds are present between 2.6A and 3.4A. Ionic interactions are displayed between 3.3A and 3.6A. The hydrophobic interactions are observed at 4.1A.The region where mutation is present is highlighted within red box that depicts the increase or decrease of molecular flexibility

**Fig. 9:**
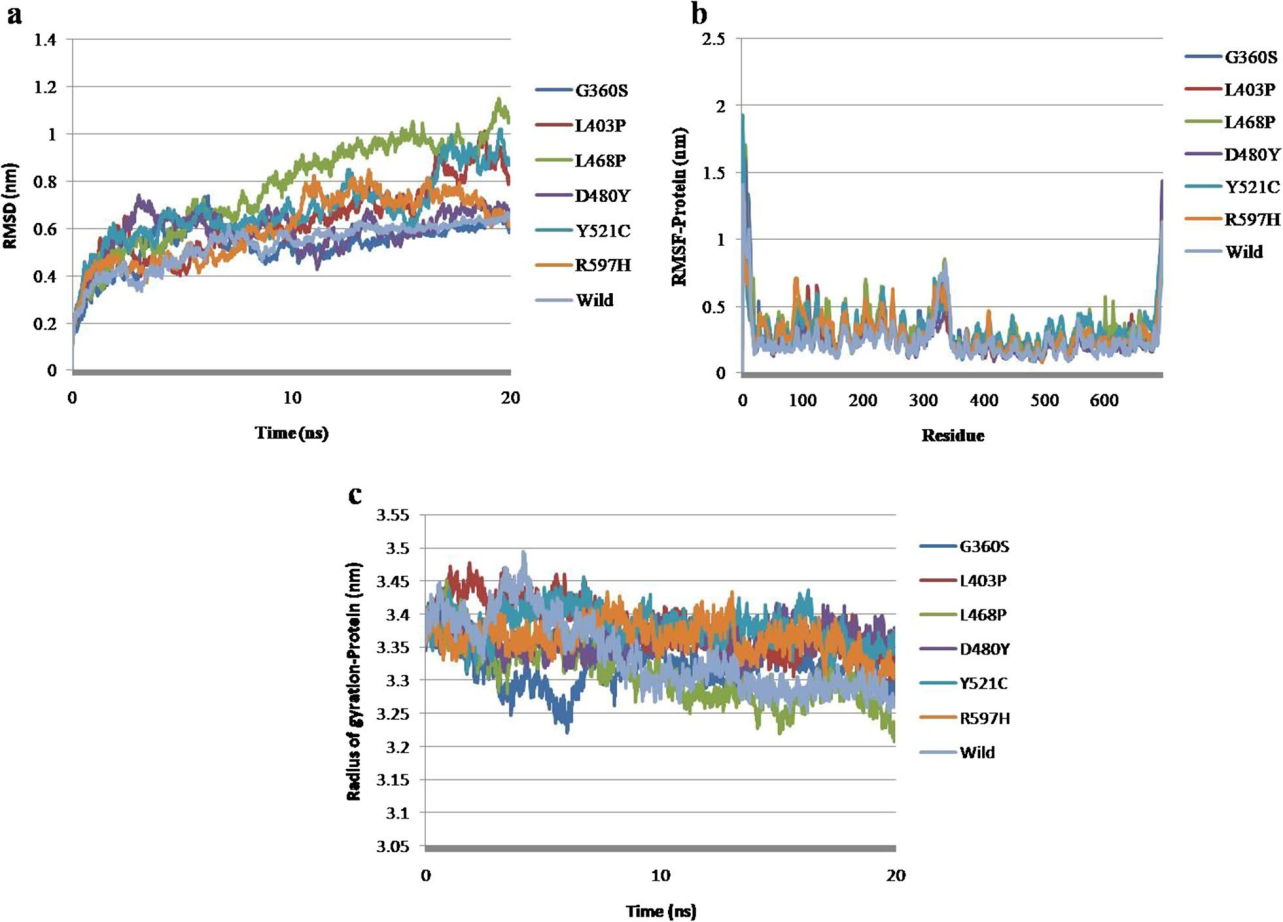
Molecular dynamics simulations representation of PRKCG nsSNPs residing in kinase domain. **a.** Root mean square deviation (RMSD). **b**. Root mean square fluctuation (RMSF)—protein. **c.** Radius of gyration (Rg)—backbone

Molecular flexibility analysis revealed that kinase domain mutations G360S, D480Y, and R597H cause a decrease in flexibility, whereas mutations of L403P, L468P, and Y521C cause flexibility to increase (Fig. 8). In mutant G360S, the loss of weak hydrogen bonds was observed while strong hydrogen bonds were retained. In mutants, L403P, L468P, and Y521C, the loss of hydrogen bonds and hydrophobic interactions has been spotted. In mutant, D480Y, the change in length and size of hydrophobic and hydrogen bonds within wild and mutant structures has been observed, while in mutant R597H, the loss of ionic interactions and gain of hydrophobic interactions have been noted. Also, a few inter-residue hydrogen bond interactions have been reduced. A molecular dynamics investigation revealed that nsSNPs caused fluctuations in the kinase region. The RMSD, RMSF, and Rg values depict instability in mutant protein structures (Fig. 9a, b, and c). Reportedly, a high Rg specifies protein structure expansion, while a low Rg shows protein structure compactness (Fig. 9c).

### Flexibility and molecular dynamics simulation analysis of mutant in C-terminal tail of PKC γ

**Fig. 10:**
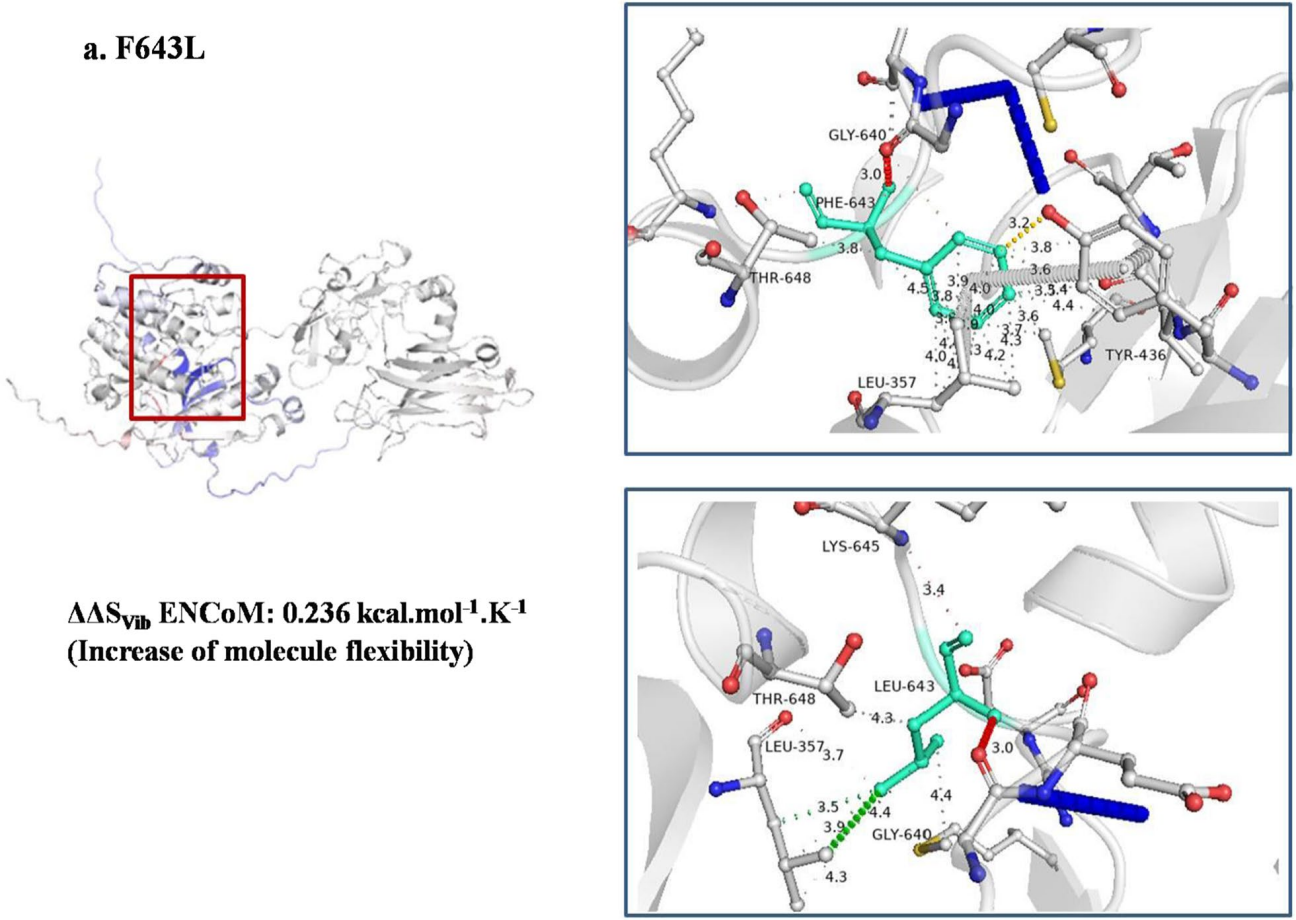
DynaMut tool representation of molecular flexibility analysis and interatomic interactions of C-terminal tail of PKC γ. **a.** Variant F643L is shown. Hydrogen bonds are present at 3.0A, 3.4A and 3.7A. Hydrophobic interactions are displayed between 3.5A and 4.5A. Ionic interactions are present at 3.2A. The region where mutation is present is highlighted within red box that depicts the increase of molecular flexibility

**Fig. 11:**
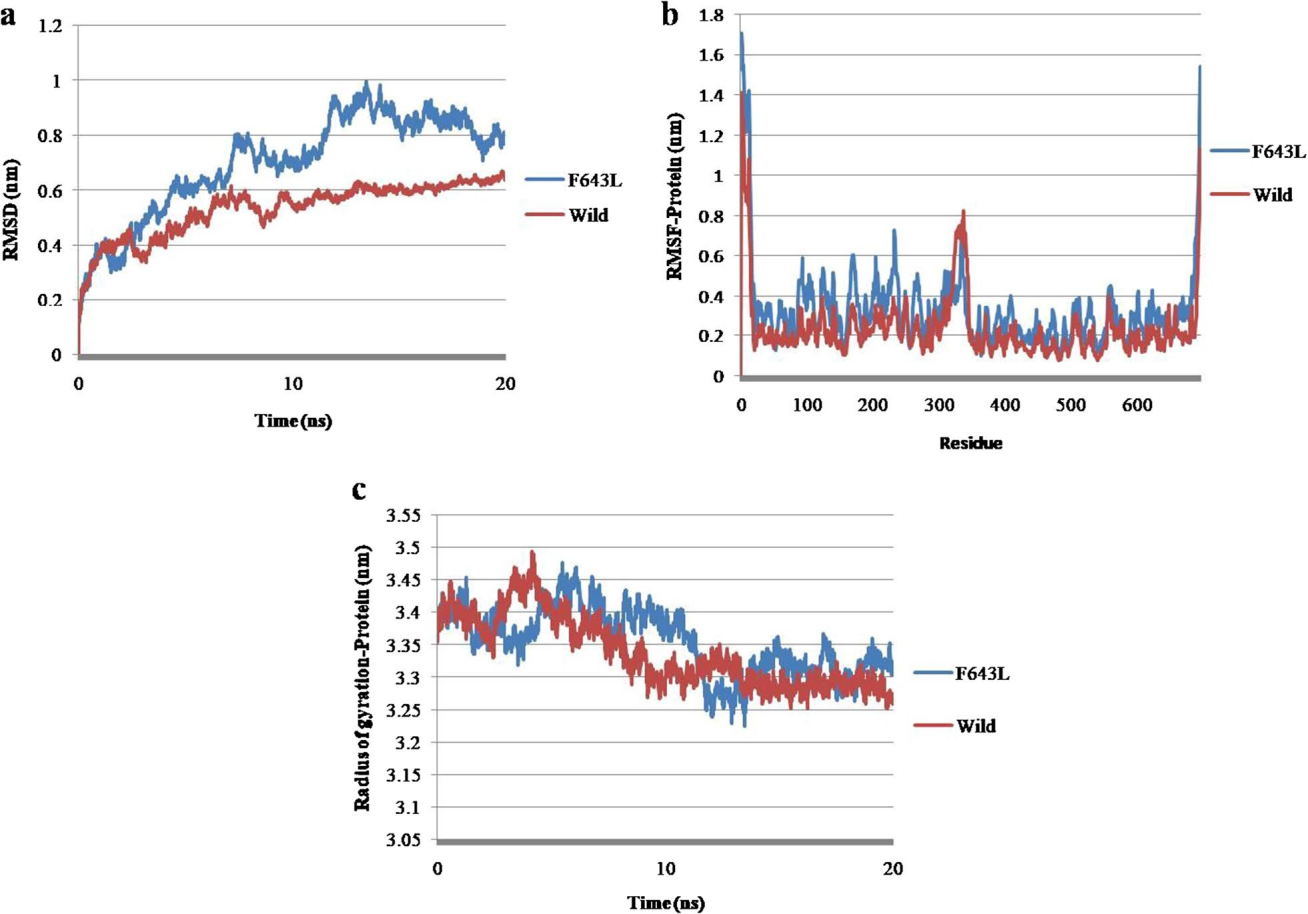
Molecular dynamics simulations representation of PRKCG nsSNP residing in C-terminal tail. **a.** Root mean square deviation (RMSD). **b**. Root mean square fluctuation (RMSF)—protein. **c.** Radius of gyration (Rg)—backbone

A single residue mutation, F643L, was identified in the C-terminal tail of PKC γ that caused an increase in molecular flexibility due to the loss of hydrophobic and ionic interactions (Fig. 10). The RMSD of the F643L variant increased in comparison with the wild type (Fig. 11a). The RMSF and Rg for protein atoms showed fluctuations throughout 20 ns (Fig. 11b, c).

### Survival rates estimation and pathological N and T stage analysis

**Fig. 12:**
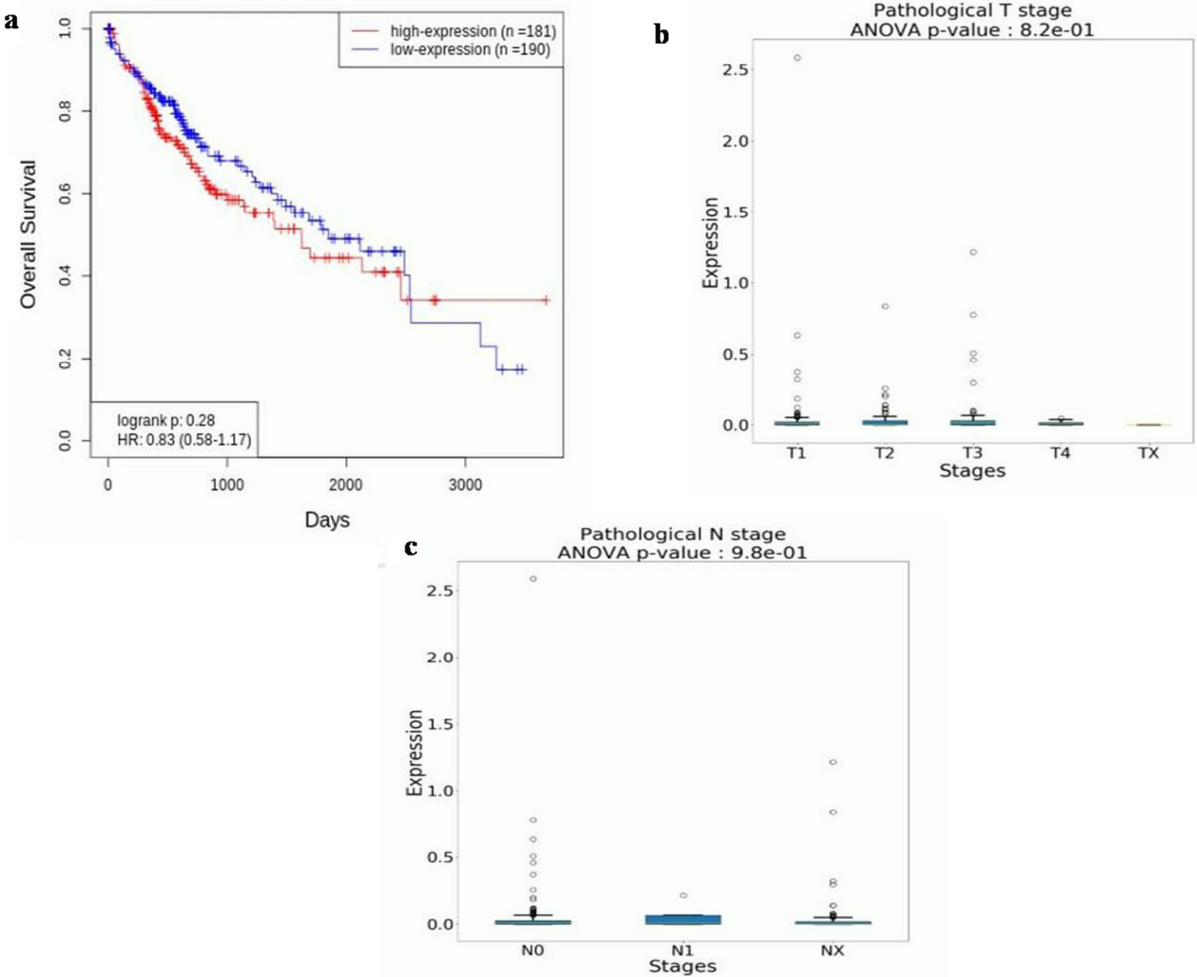
**a**. Kaplan–Meier plot showing the relationship between PRKCG levels and patient survival in liver cancer. **b**. PRKCG expression at Pathological T stages **c**. PRKCG expression at Pathological N stages

The plot of the Kaplan–Meier estimator shows that as the expression of PRKCG increases (depicted by the red line in a series of declining horizontal steps), the survival rate of liver cancer patients decreases (Fig. 12a). For the survival plot, Threshold: Cutoff was selected as ≥ 50%. In total, information related to 371 (n) patients was extracted from OncoDB. High expression of PRKCG was recorded in 181 (n) patients, while low expression was reported in 190 (n) patients. It shows that the over expression of PRKCG is hazardous for cancer patients and lowers their survival rates. Moreover, the results depict that PRKCG expression is higher during different T and N stages according to ANOVA p values, which highlights its role in metastasis (Fig. 12b, c).

### Frequency analysis of PKC γ nsSNPrs386134171 genotype (G/A) in patients and controls

In total, 100 HCC patients and 100 controls were included in the study. Through analyzing the locus rs386134171, notable differences between the frequencies of homozygous (GG, AA) and heterozygous (AG) genotypes were observed between patients (Additional file 1) and control groups (Additional file 1). GG was found in 56 controls and in 36 controls. AA was noted in 61 patients and 39 controls, while the AG genotype was identified in 3 patients and 5 controls. The GG genotype was more prevalent in controls compared to the AA genotype. AG had almost equal frequency in the studied groups. Genotype AA had more prominence in patients with OR (2.446), RR (1.564), and P-values (< 0.0029) indicating the probability that it may act as a predominant factor towards HCC onset, while the GG genotype was found to be rather protective in terms of association with HCC (Table 9).

**Table 9:** Comparison of PKC gamma genotype (G/A) frequencies in patients and control groups

Gene	Locus	Genotypes	Patients (n)	Control (n)	OR	OR (95% CI)	RR	RR (95% CI)	P-value
PRKCG	rs386134171	GG	36	56	0.4420	(0.2524, 0.7858)	0.6603	(0.4848, 0.8822)	< 0.0069
		AA	61	39	2.446	(1.377, 4.289)	1.564	(1.177, 2.109)	< 0.0029
		AG	3	5	0.5876	(0.1525, 2.308)	0.7423	(0.2687, 1.410)	> 0.7209

### Gender specific frequency analysis of PKC γ nsSNPrs386134171 genotype (G/A)

When genotype frequency comparison was done gender specifically, it was noticed that males in comparison to females had higher frequencies of the examined genotypes, GG, AA, and AG. In males, 44 controls and 22 patients had the GG genotype, 31 patients and 29 controls had the AA genotype, and 2 patients and 4 controls had the AG genotype. In females, 14 patients and 12 controls had GG genotypes, 30 patients and 10 controls had AA genotypes, and heterozygous genotype (AG) was almost equal in the patient and control groups. According to P values, a non-significant association was found between the respective genotypes and HCC (Table 10).

**Table 10:** Comparison of PKC gamma genotype (G/A) frequencies between males and females of patient and control groups

Genotypes	Patients (%)	Control (%)	OR	OR (95% CI)	RR	RR (95% CI)	P-value
GG (M)	22	44	0.50	0.2540–1.00	0.6667	0.4353–1.004	> 0.0771
AA (M)	31	29	2.138	1.064–4.235	1.550	1.035–2.343	> 0.0507
AG (M)	2	4	0.6887	0.1277–3.054	0.7925	0.2271–1.748	> 0.9999
GG (F)	14	12	0.4140	0.1509–1.126	0.7295	0.4668–1.048	> 0.1166
AA (F)	30	10	2.60	0.8774–7.231	1.40	0.9788–2.156	> 0.0755
AG (F)	1	1	0.50	0.02581–9.900	0.75	0.1410–1.435	> 0.9999

### Expression analysis

**Fig. 13:**
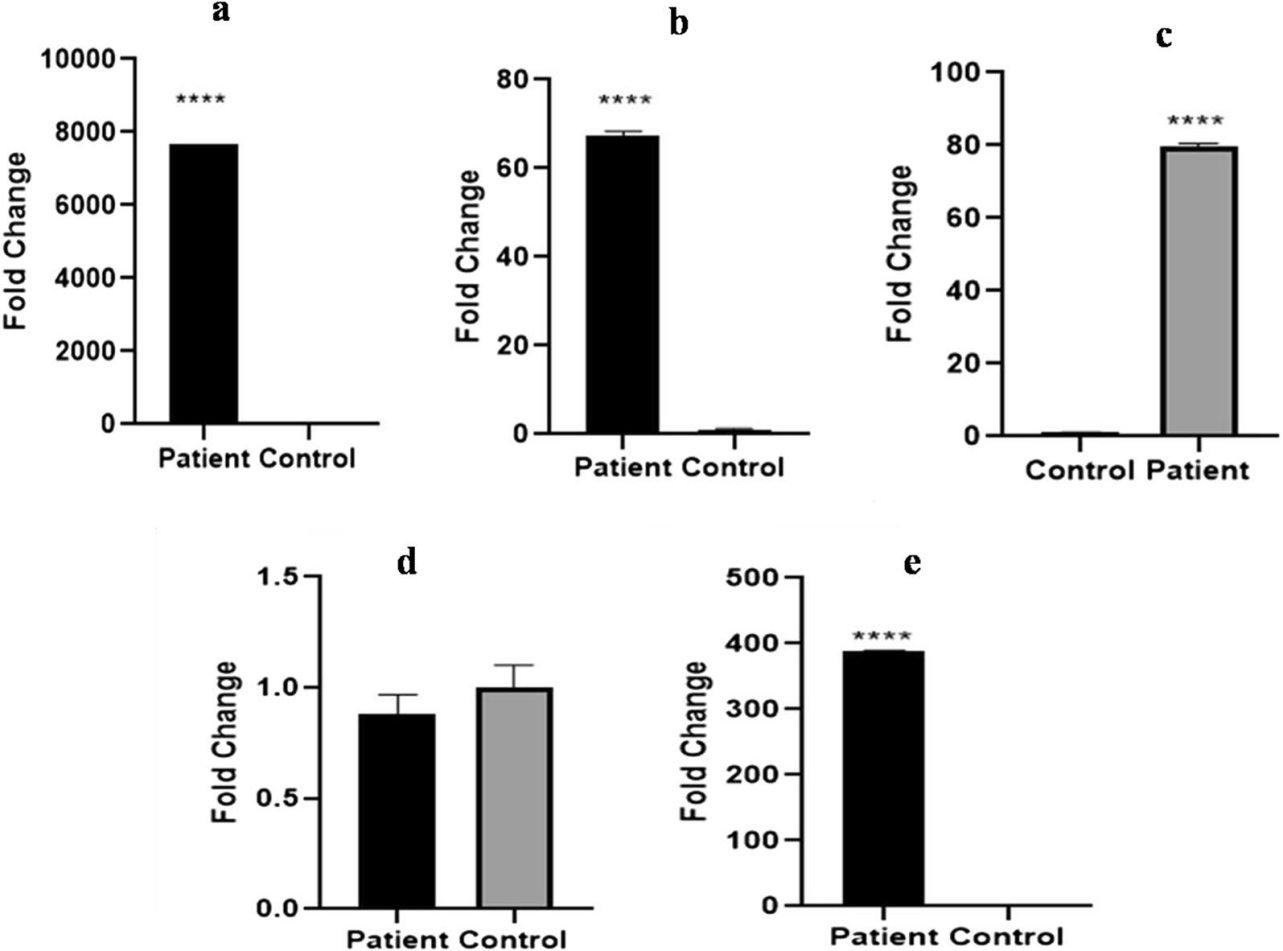
RT-PCR analysis of PRKCG, HIF1 *α,* AKT, SOCS3 and VEGF expression in blood of hepatocellular carcinoma patients. Difference in expression between patients and controls is represented in the form of fold change: **a** high expression of PKC gamma **b** high expression of HIF1*α* **c** high expression of AKT **d** low expression of SOCS3 **e** high expression of VEGF

The blood expression analysis of PKC γ, HIF1 *α,* AKT, SOCS3, and VEGF was done to analyze the fold change difference among patients and controls. Apart from SOCS3, all other genes had high expression in HCC patients, which indicates their prominent role in carcinogenesis. Among the genes analyzed, PRKCG showed a high fold change difference (Fig. 13).

### Inter-connection between PKC γ, HIF-1 alpha*,* AKT, SOCS3 and VEGF

**Fig. 14:**
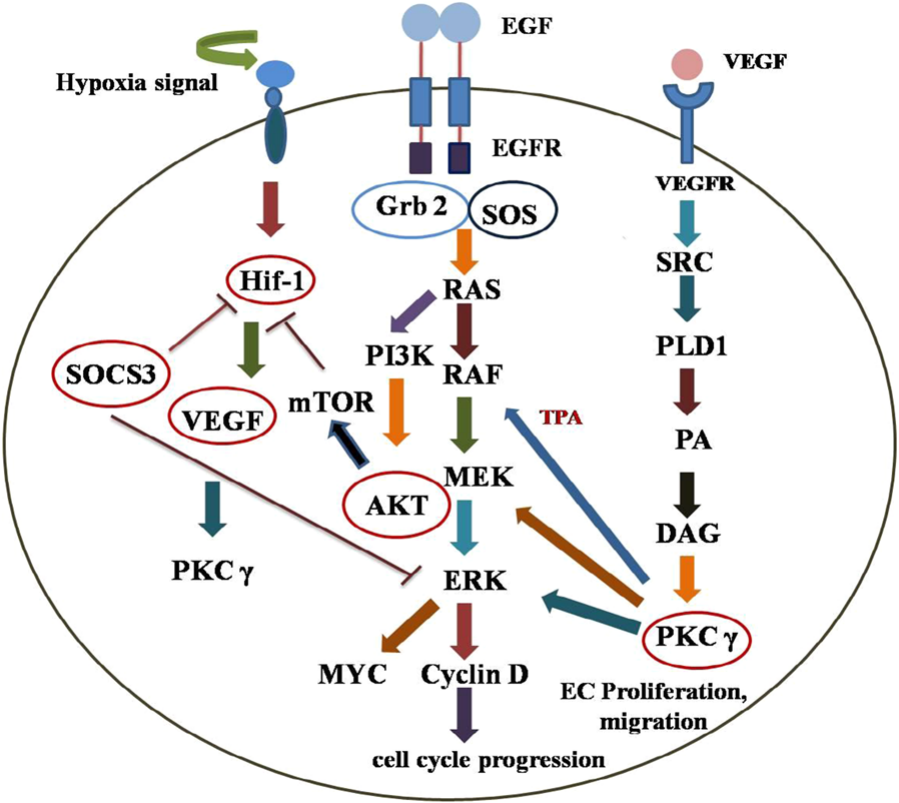
Schematic pathway representing the connection between PRKCG with other major genes involved in carcinogenesis. The gene expression levels of oncogenes and corresponding metabolites **a** PKC **γ** (**b**). Hif-1 alpha (**c**) VEGF (**d**) AKT (**e**) RAS (**f**) RAF (**g**) MEK (**h**) ERK (**i**) Cyclin D (**j**) SRC (**k**) SOS (**l**). VEGFR (**m**) Grb (**n**). EGF (**o**) EGFR (**p**) PA (**q**) PLD1 (**r**) DAG (**s**) PI3K and (**t**) TPA were found upregulated (↑) while tumor suppressor genes (u) mTOR and (v) SOCS3 were found downregulated (↓)

PKC γ, HIF-1 alpha*,*AKT, SOCS3, and VEGF expressions connect with one another in defined and recognizable pathways. HIF-1 alpha expression leads to upregulated VEGF expression, which in turn activates PKC γ. VEGF, once released, can lead to angiogenesis through binding with VEGFR, which activates SRC, which ends up with PLD1-PA/DAG/PKCγ pathway stimulation. Interestingly,PKC γ induces Raf and MEK activation via phosphorylation through the tumor promoting molecule, TPA (12-O-tetradecanoylphorbol 13-acetate). Apart from Raf and MEK, PKCγ can interact with ERK which triggers cancer cell proliferation through many proteins, including cyclin D and MYC, to name a few (Fig. 14). In cancer, SOCS3 acts as a suppressor. It can inhibit the HIF-1 alpha and ERK pathways that cross-link with PKC γ. So, indirectly, SOCS3 obstructs pathways that lead to PKC γ activation or those pathways that are controlled by PKC γ. One of the pathways that activates RAS is when mitogen factors, e.g., EGF, bind to the EGFR receptor, which in response activates Grb2, which turns on SOS activity, which acts as a guanine nucleotide exchange factor for RAS. RAS through PI3K activates AKT, which switches on HIF-1 alpha activity. So, indirectly, through upregulation of AKT, PKC γ activity is also triggered (Fig. 14).

## Discussion

Liver cancer etiology differs geographically. In other words, it is heterogeneous. HCC is deemed as the liver cancer common type and is ranked at the sixth number for its incidence rate among cancers [54, 55]. Disease susceptibility differs from one individual to another on a genetic basis that includes SNPs. Among SNPs, non-synonymous ones contribute to altered phenotype [56]. Based on PRKCG, the literature available on nsSNPs association with cancer is very limited and not studied in detail. The present study has taken the help of bioinformatics approaches before wet lab experiment validation to unravel the nsSNPs of PRKCG that could potentially be involved in carcinogenesis and are often missed for their significance in disease pathogenicity. The different filtration and sorting bioinformatics tools have been used previously in different studies, and their precise and accurate predictions have proven successful in determining whether the nsSNPs are pathogenic/deleterious, or benign [57].

The following study concluded that 15 nsSNPs were pathogenic according to SIFT, PolyPhen-2.0, Mutation Assessor, CADD, REVEL, MetaLR, FATHMM and PROVEAN scores [23, 25].

The majority of 15 nsSNP mutations were identified in exons 5, 10, and 18 and the regulatory and kinase domains of PKC γ. Structural analysis of selected high-risk nsSNPs showed that the amino acid residue substitutions in PKC γ domains had a deleterious impact on the stability and, subsequently, the function of PKC γ. Amino acid residues that had undergone substitution were rs797045900 (C52S), rs386134162 (C114Y), rs1064797249 (D115Y), rs1568752939 (G128R), rs386134167 (C131Y), rs1192424800 (V138M), rs1599943341 (C150R), rs866406014 (G159R), rs386134171 (G360S), rs747522330 (L403P), rs1398783758 (L468P), rs387906679 (D480Y), rs1202130595 (Y521C), rs1568764306 (R597H), and rs121918516 (F643L). Because of the presence of these high-risk nsSNPs, it is more likely that humans with these nsSNPs in their genome may develop different phenotypes due to altered PKC γ gene expression. Our research suggests that these nsSNPs be considered when determining the risk of several diseases. Six variant residues were found in the C1B area: one in the C1A region, one in the region between C1B and C2, six in the C3/C4 region, and one in the C-terminal tail. Studies suggest that the connection between the pseudosubstrate and catalytic domains appears to be strengthened by each regulatory domain, including C1a, C1b, and C2. Additionally, mutations that alter the C1a and other regulatory domain contacts can loosen the autoinhibited interaction, causing the enzymatic activity to increase [58].

Also, C1B domain mutations prevent the binding of secondary messengers, leading to decreased activity [16]. Similarly, mutagenesis of the C2 domain puts PKCs in an inactive conformation or lock state by developing intramolecular interactions with the catalytic domain [59]. Moreover, kinase loops, or C3/C4 domains, are the sites for phosphorylation, and mutations at these conserved sites do harm the full activation of PKC enzymes [60]. C-terminal tail facilities PKC stability and in facilitating the catalytic core interaction with ATP or the substrate [61]. The conservation scores and the disruption in the underlying molecular pathways were both determined in the subsequent study using MutPred2. Utilizing MutPred2, pathogenic mutations with underlying functional changes have been documented [62]. Additionally, the Project Hope software's findings have offered crucial details on the potential consequences of missense SNPs. Once transformed into different residues, those residues that have a very flexible wild-type structure generate perturbed folding interactions. More bumps and repulsions were induced in the structures where the mutant residue was bigger. A difference in charges and sizes between the wild-type and mutant amino acids was also noted, which disrupted the inter-molecular interactions as a result of point mutations. Additionally, it was shown that the mutant residue imparted differential charges to a buried residue, which caused issues with protein folding. As mutations took place, the torsion angles also altered, which drove the local backbone into a misaligned conformation, disrupting the local structure. It was observed that the new residues were not in the proper location to form the same intermolecular bonds as the original wild-type residue due to size and ionic discrepancies. The formation of hydrogen bonds was also impacted by the variation in hydrophobicity levels caused by changed residues.

Importantly, SNPs that form part of the conserved region of the protein, if gets mutated, have a deleterious effect compared to those that lie in the non-conserved region [63]. ConSurf software was therefore employed for determining the conservation status of amino acids. The ConSurf software was employed to give the conservation scores of amino acids by analyzing the evolutionary dynamics of homologous sequences. The exposed residues G360, D480, Y521, and R597, according to ConSurf, highlight their potential functional role in initiating various molecular pathways through surface-molecule interaction. The remaining residues, on the other hand, were found buried within the structure, indicating their structural role. From the ConSurf results, it can be deduced that those residues that were conserved when they got mutated directly impacted the structural conformation and stability [63].

DynaMut2 was also used in the study, which incorporates information on protein dynamics and structural environment properties of wild‐type residues and proteins with single and multiple point mutations [64]. The results of the DynaMut2 software tool helped in the identification of changes in hydrogen, hydrophobic, ionic, and other electrostatic bond interactions in residues from wild-type to mutant types. A molecular dynamics investigation revealed that nsSNPs caused fluctuations in the kinase, regulatory, and C-terminal regions. Moreover, the RMSD, RMSF, and Rg values depict instability in mutant protein structures. The molecular dynamic simulation was run for 20 ns, if it is expanded to 50 ns or more, as indicated in past studies, it can give more accurate insight into the PKC γ molecular dynamics. [65]. This is primarily the first study that has linked the decreased survival rates of liver cancer patients with the over expression of PRKCG through Kaplan Meier Analysis. The pathological stages signify that as PRKCG expression has been upregulated, the tumor has become more aggressive and invasive. Moreover, blood expression analyses have highlighted upregulated and downregulated genes in HCC. Apart from SOCS3, all other genes studied had high expression that specify towards their prominent role in carcinogenesis. In the current study, a genetic cascade was constructed that depict PKC γ pathway interactions with other molecules in biochemical pathways. PKC γ is activated downstream of the HIF-1 alpha/VEGF pathway, which gets activated during hypoxic conditions when cancer cells are proliferating and demand and supply of oxygen do not fulfill their need for angiogenesis. [66]. Interestingly, the research has pointed out that apart from Ras GTP that switch on Raf/MEK/ERK pathway, PKC γ induces Raf and MEK activation via phosphorylation through tumor promoting molecule, TPA (12-O-tetradecanoyl phorbol 13-acetate) [67, 68]. Apart from Raf and MEK, PKCγ can interact with ERKthat triggers cancer cells proliferation through cyclin D1 [69].In cancer, SOCS3 acts as a suppressor. It can inhibit HIF-1 alpha and ERKpathway that cross-links with PKC γ [70, 71]. AKT upregulate HIF 1 alpha levels [72] that triggers PKC γ activation.

In the present study, genotyping analysis was done gender specifically among patients and controls to notice the association of nsSNP (rs386134171) with HCC. The mutant genotype AA (homozygous) had more frequency in patients compared to controls, while the wild genotype GG had more frequency in controls. The heterozygous genotype AG showed no significant relation with disease onset, and the heterozygous genotype was almost equal in the patient and control groups. The results of the current study are coherent with the previous studies, signifying the role of variant alleles in disease onset [73].

As the SNP is non-synonymous, it has a direct impact on the phenotype of the resulting protein. So, through the investigation, it has been confirmed that the variant alleles have played a key role in altering the activity of PKC γ, which in turn can change its interaction pattern with other proteins. The current study raises questions regarding tumor relapse, metastasis, and chemoresistance in patients with HCC and pinpoints the significance of nsSNP in tumor progression that ultimately leads to aggressive tumors and treatment failure. The studied nsSNP, rs386134171, can act as a solid genetic marker in HCC diagnosis.

## Conclusion

The specific function of PKC γ in HCC remains largely unknown. Moreover, there is a need to conduct a detailed study on HCC so that sufficient literature on it is available. Through current study, PKC γ role in liver cancer has become evident. This is a major study in terms of its uniqueness because it has provided a detailed list of pathogenic nsSNPs most likely contributing to cancer onset. Through wet-lab analysis, the involvement of one of the nsSNPs of PRKCG, i.e., rs386134171 in HCC progression hasbeen confirmed. This study has without a doubt set a direction for future studies that focus on finding novel genetic markers for HCC.

## Supplementary Information

**Additional file 1: **Non-synonymous SNPs variants of PRKCG and its association with oncogenes predispose to hepatocellular carcinoma.

## Data Availability

Data as supplementary material is provided along with the manuscript. Raw data will be available from corresponding author on request.
