# Supplementary material for: Non-synonymous SNPs variants of PRKCG and its association with oncogenes predispose to hepatocellular carcinoma
Source: Cancer Cell Int. 2023 Jun 21;23:123. doi: 10.1186/s12935-023-02965-z (PMC10286404; doi:10.1186/s12935-023-02965-z)
Supplement: Supplementary file 1 — Additional file 1: Non-synonymous SNPs variants of PRKCG and its association with oncogenes predispose to hepatocellular carcinoma. [file 12935_2023_2965_MOESM1_ESM.pdf]

Supplementary File S1

Non-synonymous SNPs variants of PRKCG and its association with oncogenes predispose to hepatocellular carcinoma

Fizzah Abid[1], Khushbukhat Khan[1], Yasmin Badshah[1], Naeem Mahmood Ashraf[2], Maria Shabbir[1], Arslan Hamid[3,] Tayyaba Afsar[4], Ali Almajwal[4,] Suhail Razak[4]

Table 1: Protein stability predictions are depicted. The values are displayed in the form of DDG (kcal/mol)

Residues	MUpro	DynaMut	i-Mutant
C52S	-1.5	0.110	0.1
C114Y	-0.969	0.929	-0.22
D115Y	-0.870	-0.102	0.37
G128R	-0.374	-0.670	0.38
C131Y	-0.440	-0.605	0.14
V138M	-0.943	-0.691	0.31
C150R	-1.65	0.417	-0.22
G159R	-0.535	0.925	-0.95
G360S	-0.696	0.859	-0.05
L403P	-1.73	-0.738	-1.74
L468P	-2.317	-1.497	-2.21
D480Y	-1.323	0.046	-0.89
Y521C	-0.604	-1.452	0.85
R597H	-0.622	-1.191	-0.04
F643L	-1.108	-1.034	-0.85

Table 2: The 427 rsIDs description as deleterious or benign

SIFT	PolyPhen-2	Mutation Assessor	MetaLR	REVEL	CADD
Deleterious: 234	Benign: 222	Low: 159	Tolerated: 344	Benign: 321	Benign: 370
Tolerated: 193	Probably Damaging: 131	Neutral: 125	Damaging: 83	Damaging: 106	Deleterious: 57
	Possibly Damaging: 74	Medium: 88			
		High: 54			

Table 3: The exon positions of PRKCG are shown along with genomic coordinates, amino acid coordinates and the number of filtered amino acid residues encoded by different exons.

Exon no.	Chr:bp (Start)	Chr:bp (End)	Amino acid coordinates	No. of Amino acids residues
Exon 1	19:53882197	19:53882664	1-56	31
Exon 2	19:53883163	19:53883194	58-66	5
Exon 3	19:53884161	19:53884243	68-95	11
Exon 4	19:53889638	19:53889749	96-131	25
Exon 5	19:53889886	19:53890017	133-176	41
Exon 6	19:53891674	19:53891830	177-228	30
Exon 7	19:53892509	19:53892643	230-273	32
Exon 8	19:53892988	19:53893075	275-300	15
Exon 9	19:53893362	19:53893391	304-312	7
Exon 10	19:53897959	19:53898111	314-361	43
Exon 11	19:53898440	19:53898628	366-427	34
Exon 12	19:53900233	19:53900324	429-458	19
Exon 13	19:53900419	19:53900481	460-477	11
Exon 14	19:53900611	19:53900749	480-521	18
Exon 15	19:53903073	19:53903153	527-541	6
Exon 16	19:53904635	19:53904742	553-588	22
Exon 17	19:53906317	19:53906457	589-635	35
Exon 18	19:53906707	19:53907652	636-696	42

Table 4: The model confidence levels of different amino acid residues as depicted by Alphafold

Amino Acid Residue Positions	Model Confidence Levels
1-20	Very low
21-28	low
29-35	Confident
36-38	Very high
39-46	Confident
47-53	Very high
54-57	Confident
58-60	low
61-63	Confident
64-66	Very high
67-68	Confident
69-74	Very high
75-76	Confident
77-78	Very high
79	Confident
80	Very High
81-88	Confident
89-90	low
91-92	Very low
93-97	low
98-101	Confident
102-120	Very high
121-126	Confident
127-132	Very high
133-134	Confident
135-140	Very high
141	Confident
142-143	Very high
144-156	Confident
157-184	Very high
185	Confident
186	Very high
187-191	Confident
192-203	Very high
204	Confident
205-232	Very high
233-234	Confident
235-247	Very high
248-252	Confident

Table 8

Patient IDs	Genotype	Homozygous/ Heterozygous	Gender
1	AA	Homozygous	Male
2	AA	Homozygous	Male
3	AA	Homozygous	Male
4	AA	Homozygous	Male
5	AA	Homozygous	Male
6	AA	Homozygous	Male
7	AA	Homozygous	Male
8	AA	Homozygous	Male
9	AA	Homozygous	Male
10	AA	Homozygous	Male
11	AA	Homozygous	Male
12	AA	Homozygous	Male
13	AA	Homozygous	Male
14	AA	Homozygous	Male
15	AA	Homozygous	Male
16	AA	Homozygous	Male
17	AA	Homozygous	Male
18	AA	Homozygous	Male
19	AA	Homozygous	Male
20	AA	Homozygous	Male
21	AA	Homozygous	Male
22	AA	Homozygous	Male
23	AA	Homozygous	Male
24	AA	Homozygous	Male
25	AA	Homozygous	Male
26	AA	Homozygous	Male
27	AA	Homozygous	Male
28	AA	Homozygous	Male
29	AA	Homozygous	Male
30	AA	Homozygous	Female
31	AA	Homozygous	Female
32	AA	Homozygous	Female
33	AA	Homozygous	Female
34	AA	Homozygous	Female
35	AA	Homozygous	Female
36	AA	Homozygous	Female
37	AA	Homozygous	Female
38	AA	Homozygous	Female
39	AA	Homozygous	Female
40	GG	Homozygous	Male
41	GG	Homozygous	Male
42	GG	Homozygous	Male
43	GG	Homozygous	Male
44	GG	Homozygous	Male
45	GG	Homozygous	Male

46	GG	Homozygous	Male
47	GG	Homozygous	Male
48	GG	Homozygous	Male
49	GG	Homozygous	Male
50	GG	Homozygous	Male
51	GG	Homozygous	Male
52	GG	Homozygous	Male
53	GG	Homozygous	Male
54	GG	Homozygous	Male
55	GG	Homozygous	Male
56	GG	Homozygous	Male
57	GG	Homozygous	Male
58	GG	Homozygous	Male
59	GG	Homozygous	Male
60	GG	Homozygous	Male
61	GG	Homozygous	Male
62	GG	Homozygous	Male
63	GG	Homozygous	Male
64	GG	Homozygous	Male
65	GG	Homozygous	Male
66	GG	Homozygous	Male
67	GG	Homozygous	Male
68	GG	Homozygous	Male
69	GG	Homozygous	Male
70	GG	Homozygous	Male
71	GG	Homozygous	Male
72	GG	Homozygous	Male
73	GG	Homozygous	Male
74	GG	Homozygous	Male
75	GG	Homozygous	Male
76	GG	Homozygous	Male
77	GG	Homozygous	Male
78	GG	Homozygous	Male
79	GG	Homozygous	Male
80	GG	Homozygous	Male
81	GG	Homozygous	Male
82	GG	Homozygous	Male
83	GG	Homozygous	Male
84	GG	Homozygous	Female
85	GG	Homozygous	Female
86	GG	Homozygous	Female
87	GG	Homozygous	Female
88	GG	Homozygous	Female
89	GG	Homozygous	Female
90	GG	Homozygous	Female
91	GG	Homozygous	Female
92	GG	Homozygous	Female

93	GG	Homozygous	Female
94	GG	Homozygous	Female
95	GG	Homozygous	Female
96	AG	Heterozygous	Male
97	AG	Heterozygous	Male
98	AG	Heterozygous	Male
99	AG	Heterozygous	Male
100	AG	Heterozygous	Female

Table 7

Patient IDs	Genotype	Homozygous/ Heterozygous	Gender
1	AA	Homozygous	Male
2	AA	Homozygous	Male
3	AA	Homozygous	Male
4	AA	Homozygous	Male
5	AA	Homozygous	Male
6	AA	Homozygous	Male
7	AA	Homozygous	Male
8	AA	Homozygous	Male
9	AA	Homozygous	Male
10	AA	Homozygous	Male
11	AA	Homozygous	Male
12	AA	Homozygous	Male
13	AA	Homozygous	Male
14	AA	Homozygous	Male
15	AA	Homozygous	Male
16	AA	Homozygous	Male
17	AA	Homozygous	Male
18	AA	Homozygous	Male
19	AA	Homozygous	Male
20	AA	Homozygous	Male
21	AA	Homozygous	Male
22	AA	Homozygous	Male
23	AA	Homozygous	Male
24	AA	Homozygous	Male
25	AA	Homozygous	Male
26	AA	Homozygous	Male
27	AA	Homozygous	Male
28	AA	Homozygous	Male
29	AA	Homozygous	Male
30	AA	Homozygous	Male
31	AA	Homozygous	Male
32	AA	Homozygous	Female
33	AA	Homozygous	Female
34	AA	Homozygous	Female
35	AA	Homozygous	Female
36	AA	Homozygous	Female
37	AA	Homozygous	Female
38	AA	Homozygous	Female
39	AA	Homozygous	Female
40	AA	Homozygous	Female
41	AA	Homozygous	Female
42	AA	Homozygous	Female
43	AA	Homozygous	Female
44	AA	Homozygous	Female
45	AA	Homozygous	Female

46	AA	Homozygous	Female
47	AA	Homozygous	Female
48	AA	Homozygous	Female
49	AA	Homozygous	Female
50	AA	Homozygous	Female
51	AA	Homozygous	Female
52	AA	Homozygous	Female
53	AA	Homozygous	Female
54	AA	Homozygous	Female
55	AA	Homozygous	Female
56	AA	Homozygous	Female
57	AA	Homozygous	Female
58	AA	Homozygous	Female
59	AA	Homozygous	Female
60	AA	Homozygous	Female
61	AA	Homozygous	Female
62	GG	Homozygous	Male
63	GG	Homozygous	Male
64	GG	Homozygous	Male
65	GG	Homozygous	Male
66	GG	Homozygous	Male
67	GG	Homozygous	Male
68	GG	Homozygous	Male
69	GG	Homozygous	Male
70	GG	Homozygous	Male
71	GG	Homozygous	Male
72	GG	Homozygous	Male
73	GG	Homozygous	Male
74	GG	Homozygous	Male
75	GG	Homozygous	Male
76	GG	Homozygous	Male
77	GG	Homozygous	Male
78	GG	Homozygous	Male
79	GG	Homozygous	Male
80	GG	Homozygous	Male
81	GG	Homozygous	Male
82	GG	Homozygous	Male
83	GG	Homozygous	Male
84	GG	Homozygous	Female
85	GG	Homozygous	Female
86	GG	Homozygous	Female
87	GG	Homozygous	Female
88	GG	Homozygous	Female
89	GG	Homozygous	Female
90	GG	Homozygous	Female
91	GG	Homozygous	Female
92	GG	Homozygous	Female

93	GG	Homozygous	Female
94	GG	Homozygous	Female
95	GG	Homozygous	Female
96	GG	Homozygous	Female
97	GG	Homozygous	Female
98	AG	Heterozygous	Male
99	AG	Heterozygous	Male
100	AG	Heterozygous	Female

Tablep

PTM1	AA1	POSITION1	CONSERVA	PTM2	AA2	POSITION2	CONSERVATION2
phosphoryl	T	512	95	acetylation	K	503	81
phosphoryl	T	512	95	ubiquitinat	K	503	81
phosphoryl	T	512	95	ubiquitinat	K	482	91
phosphoryl	T	512	95	phosphoryl	T	655	90
phosphoryl	T	512	95	ubiquitinat	K	587	91
phosphoryl	T	512	95	acetylation	K	587	81
phosphoryl	T	512	95	phosphoryl	T	518	98
phosphoryl	T	512	95	phosphoryl	T	511	98
phosphoryl	T	512	95	phosphoryl	Y	532	97
phosphoryl	T	512	95	phosphoryl	T	514	98
phosphoryl	T	512	95	ubiquitinat	K	596	84
phosphoryl	T	512	95	phosphoryl	Y	529	83
phosphoryl	T	512	95	ubiquitinat	K	645	84
phosphoryl	T	512	95	acetylation	K	645	84
phosphoryl	T	512	95	phosphoryl	Y	521	83
phosphoryl	T	512	95	acetylation	K	197	92
phosphoryl	T	512	95	ubiquitinat	K	197	92
phosphoryl	T	512	95	acetylation	K	359	71
phosphoryl	T	512	95	ubiquitinat	K	592	84
phosphoryl	T	176	77	phosphoryl	S	687	82
phosphoryl	T	176	77	phosphoryl	S	148	89
phosphoryl	T	176	77	ubiquitinat	K	216	51
phosphoryl	T	47	88	acetylation	K	503	81
phosphoryl	T	47	88	ubiquitinat	K	503	81
phosphoryl	T	47	88	ubiquitinat	K	482	91
phosphoryl	T	47	88	ubiquitinat	K	44	82
phosphoryl	S	687	82	acetylation	K	503	81
phosphoryl	S	687	82	ubiquitinat	K	503	81
phosphoryl	S	687	82	phosphoryl	Y	285	58
phosphoryl	S	687	82	phosphoryl	S	320	74
phosphoryl	S	687	82	ubiquitinat	K	482	91
phosphoryl	S	687	82	phosphoryl	Y	108	80
phosphoryl	S	687	82	O-linked gl	S	639	84
phosphoryl	S	687	82	O-GlcNAc g	S	639	84
phosphoryl	S	687	82	phosphoryl	S	639	84
phosphoryl	S	687	82	phosphoryl	S	11	90
phosphoryl	S	687	82	phosphoryl	S	342	90
phosphoryl	S	687	82	phosphoryl	Y	675	62
phosphoryl	S	687	82	phosphoryl	S	322	78
phosphoryl	S	687	82	O-linked gl	S	326	75
phosphoryl	S	687	82	O-GlcNAc g	S	326	75
phosphoryl	S	687	82	phosphoryl	S	326	75
phosphoryl	S	687	82	O-linked gl	S	328	86
phosphoryl	S	687	82	O-GlcNAc g	S	328	86
phosphoryl	S	687	82	phosphoryl	S	328	86

phosphoryl	S	687	82	phosphoryl	Y	195	83
phosphoryl	S	687	82	phosphoryl	Y	307	47
phosphoryl	S	687	82	phosphoryl	Y	286	83
phosphoryl	S	687	82	O-linked gl	S	321	81
phosphoryl	S	687	82	O-GlcNAc g	S	321	81
phosphoryl	S	687	82	phosphoryl	S	321	81
phosphoryl	S	687	82	O-linked gl	S	690	79
phosphoryl	S	687	82	O-GlcNAc g	S	690	79
phosphoryl	S	687	82	phosphoryl	S	690	79
phosphoryl	S	687	82	phosphoryl	S	373	86
phosphoryl	S	687	82	phosphoryl	Y	532	97
phosphoryl	S	687	82	phosphoryl	S	148	89
phosphoryl	S	687	82	ubiquitinat	K	596	84
phosphoryl	S	687	82	phosphoryl	Y	529	83
phosphoryl	S	687	82	ubiquitinat	K	645	84
phosphoryl	S	687	82	acetylation	K	645	84
phosphoryl	S	687	82	phosphoryl	Y	275	56
phosphoryl	S	687	82	acetylation	K	199	92
phosphoryl	S	687	82	ubiquitinat	K	232	75
phosphoryl	S	687	82	ubiquitinat	K	216	51
phosphoryl	S	687	82	phosphoryl	T	332	77
phosphoryl	S	687	82	phosphoryl	Y	521	83
phosphoryl	S	687	82	phosphoryl	Y	312	60
phosphoryl	S	687	82	acetylation	K	197	92
phosphoryl	S	687	82	ubiquitinat	K	197	92
phosphoryl	S	687	82	O-linked gl	S	330	85
phosphoryl	S	687	82	O-GlcNAc g	S	330	85
phosphoryl	S	687	82	phosphoryl	S	330	85
phosphoryl	S	687	82	acetylation	K	359	71
phosphoryl	S	687	82	ubiquitinat	K	44	82
phosphoryl	S	687	82	ubiquitinat	K	592	84
phosphoryl	S	687	82	ubiquitinat	K	335	76
phosphoryl	T	648	90	phosphoryl	Y	675	62
phosphoryl	T	648	90	O-linked gl	T	591	84
phosphoryl	T	648	90	O-GlcNAc g	T	591	84
phosphoryl	T	648	90	ubiquitinat	K	596	84
phosphoryl	T	648	90	ubiquitinat	K	645	84
phosphoryl	T	648	90	acetylation	K	645	84
phosphoryl	T	648	90	ubiquitinat	K	232	75
phosphoryl	T	648	90	ubiquitinat	K	592	84
phosphoryl	T	648	90	O-linked gl	T	82	83
phosphoryl	T	648	90	O-GlcNAc g	T	82	83
phosphoryl	T	648	90	ubiquitinat	K	335	76
O-linked gl	T	396	87	ubiquitinat	K	400	67
O-GlcNAc g	T	396	87	ubiquitinat	K	400	67
acetylation	K	503	81	phosphoryl	S	70	86
ubiquitinat	K	503	81	phosphoryl	S	70	86

253-289	Very high
290	Confident
291-292	low
293-343	Very low
344-345	low
346-350	Confident
351-353	Very high
354-359	Confident
360-361	low
362-364	Confident
365-370	Very high
371-374	Confident
375-382	Very high
383-388	Confident
389	low
390-395	Confident
396-405	Very high
406	Confident
407-411	low
412-413	Confident
414-426	Very high
427-428	Confident
429-438	Very high
439-440	Confident
441-442	Very high
443-444	Confident
445	Very high
446-450	Confident
451-507	Very high
508-509	Confident
510-512	Very high
513-520	Confident
521-526	Very high
527-530	Confident
531-550	Very high
551	Confident
552-555	Very high
556-557	Confident
558	low
559-566	Confident
567	Very high
568-569	confident
570-599	Very high
600-603	Confident
604-630	Very high

631-634	Confident
635-636	low
637-657	Confident
658	low
659	Confident
660-664	low
665-669	Confident
670	Very high
671-672	Confident
673-677	Very high
678-684	Confident
685-686	low
687-697	Very low
